# Non-Catalyzed Cascade Double Imination Reaction of 2‑Fluoro-alk-3-yn-1-ones: Sustainable Synthesis of 3‑Fluoro-1,5-benzodiazepines

**DOI:** 10.1021/acsorginorgau.5c00116

**Published:** 2026-01-30

**Authors:** Trevor L. Olson, Ariela W. Kaspi-Kaneti, Adrian Zając, Dominic Agyei Gyimah, Austin Walsh, Jacob A. Weston, Alexander A. Rusakov, Kraig A. Wheeler, Béla Török, Roman Dembinski

**Affiliations:** † Department of Chemistry, 6918 Oakland University, 146 Library Drive, Rochester, Michigan 48309-4479, United States; ‡ Centre of Molecular and Macromolecular Studies, Polish Academy of Sciences, Sienkiewicza 112, 90-363 Łódź, Poland; § Department of Chemistry, 7448 Whitworth University, 300 W. Hawthorne Rd., Spokane, Washington 99251, United States; ∥ Department of Chemistry, University of Massachusetts Boston, 100 Morrissey Blvd., Boston, Massachusetts 02125, United States

**Keywords:** fluorine, benzodiazepines, ^19^F NMR, alkynes, catalyst-free, DFT calculations, mechanism

## Abstract

The cascade reactions of 2-fluoroalk-3-yn-1-ones with *o*-phenylenediamines provide an effective synthetic method with high atom economy for the preparation of diversely substituted 3-fluoro-3*H*-benzo­[*b*]­[1,4]­diazepines (52–81%). This noncatalytic and sustainable process embraces formal hydroamination and imination reactions, resulting in the formation of two C=N and two C–H bonds. DFT modeling provided additional insight into the reaction’s mechanism, favoring the pathway involving initial cyclization of nonconjugated enaminone, *o*-aminoanilino-2-fluorobut-3-en-1-one. The reaction may be diverted to the formation of conjugated enaminone (*o*-aminoanilino-2-fluorobut-2-en-1-one), whose presence was confirmed by ^1^H NMR. The conformational properties of the 7-membered ring were investigated by the ^1^H and ^19^F variable temperature NMR studies in solution and by the X-ray structure determination of 3-fluoro-4-(4-methylbenzyl)-2-phenyl-3*H*-benzo­[*b*]­[1,4]­diazepine.

## Graphical abstract

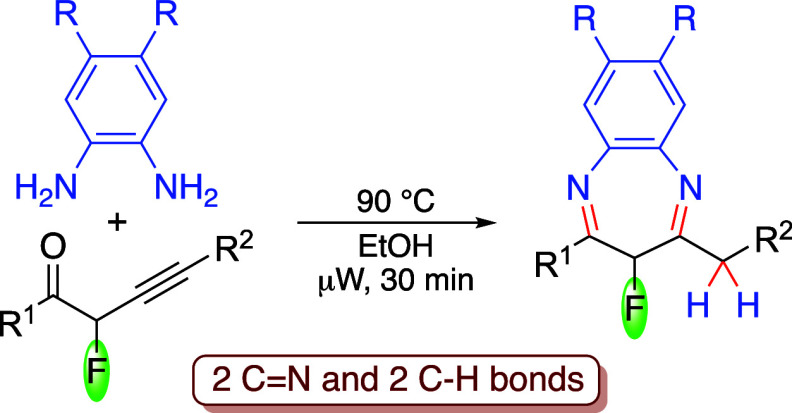

## Introduction

The incorporation of fluorine into active pharmaceutical ingredients has demonstrated the propensity to enhance their properties and offers advances in the treatment of various diseases.^1,2^ Fluorine-containing compounds occupy top-tier positions on the list of FDA-approved drugs, and a substantial amount of the most successful drugs contain fluorine atoms.^3,4^ Procedures describing divergent syntheses targeting the introduction of fluorine into privileged scaffolds, such as benzodiazepines, with a high atom economy are of interest to the synthetic organic community. Thus, developing routes leading to fluorinated heterocyclic compounds from readily available starting materials is an important objective.

Benzodiazepines^5^ are commonly used in the pharmaceutical industry,^6^ displaying a wide range of biological effects, and represent a relatively substantial number of entries on the current list of drugs.^7^ This extensive array of substances offers a broad spectrum of pharmaceutical activity due to their diverse structures. The typical pharmacological effects of benzodiazepines arise from their interaction with γ-aminobutyric acid (GABA_A_) receptors in the human brain.^8,9^ Since new benzodiazepines may bring enhanced efficacy with fewer side effects, the observed synthetic versatility in benzodiazepines offers an attractive approach for exploring unique scaffolds.

Active derivatives of 1,5-benzodiazepines, with two nitrogen atoms bonded to an aromatic ring, provide a common framework for the widely prescribed antioxidant and anticonvulsant medication Clobazam, as well as Arfendazam, Clozapine, Olanzapine, and Lofendazam (Figure 1). Since some benzodiazepines carry detrimental psychotropic and other side effects,^10^ there is a critical need to expand the current range of available compounds. Due to the potential pharmaceutical impact or the need to develop new functionalities for materials chemistry,^11,12^ novel synthetic methods for accessing expansive classes of benzodiazepines are of considerable interest.^13,14^

**Figure 1:**
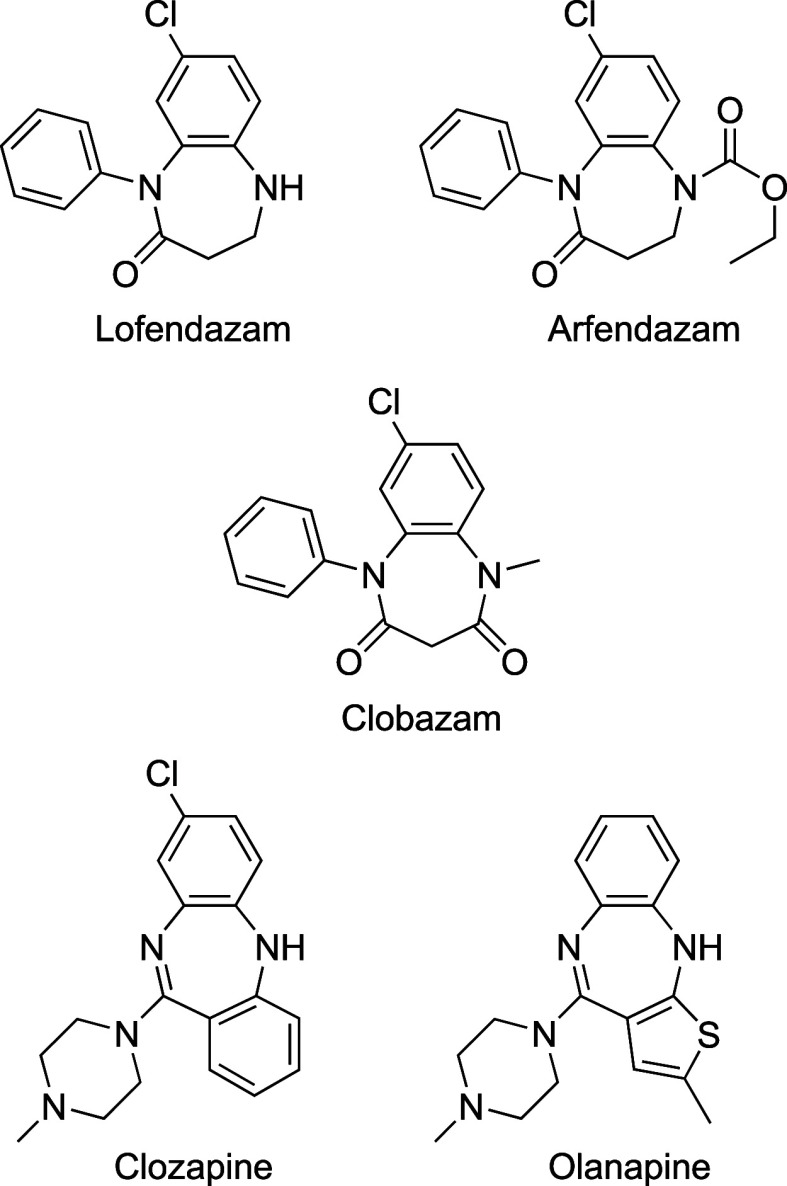
Representative therapeutically useful 1,5-benzodiazepines.

Synthetic protocols leading to 3-*mono*fluoro-1,5-benzodiazepine structures [3-fluoro-3*H*-benzo­[*b*]­[1,4]­diazepines] lack atom economy and are typically limited to specifically C-2 and C-4 functionalized scaffolds (Figure 2). Syntheses utilizing mainly 1,3-dicarbonyl diesters and esters provide access to molecular motifs with up to two carbonyl groups embedded into a seven-membered ring at the C-2 and/or C-4 position (amide functionality).^15–17^ More specifically, 2,4-bis­(dimethylamino)-3-fluoro-1,5-benzodiazepines have been prepared from phosgeniminium chloride.^18^ The route to trifluoromethyl 3-fluoro-1,5-benzodiazepine derivatives includes gallium­(III) triflate-catalyzed reaction of diamines with heptafluoropentane-2,4-dione in methylene chloride at 100 °C (Figure 2a).^19^ Perfluorinated 1-aryl-1-(trialkylsilyl)­alkanol, 1-alkyl-1-(trialkylsilyloxy)­alk-1-ene, or 2,3-difluoro-2-alkenone react with *o*-phenylenediamine in the presence of triethylamine (to eliminate hydrogen fluoride), leading to 2-perfluorobutyl or 2-perfluoroethyl 3-fluoro-1,5-benzodiazepines (Figure 2b).^20,21^ Fluorination of α-oxoketene *N*,*S*-acetals yields a mixture of compounds, which subsequently react with *o*-phenylenediamines in the presence of triethylamine (120 °C, toluene, 24 h), leading to 2-arylamino-3-fluoro-1,5-benzodiazepines (Figure 2c).^22^ Considering that the reactivity patterns of the two amino groups may differ, the synthetic strategy of the present work includes reactions of *o*-phenylenediamines (1,2-diaminobenzenes) with 2-fluoroalk-3-yn-1-ones (Figure 2d). Using a bifunctional reagent results in a chemoselective cascade process, where the amino group reacts first with an alkyne, followed by an intramolecular process that engages the second amino group and a ketone, creating the potential for the incorporation of nonsymmetrically decorated *o*-phenylenediamines.

**Figure 2:**
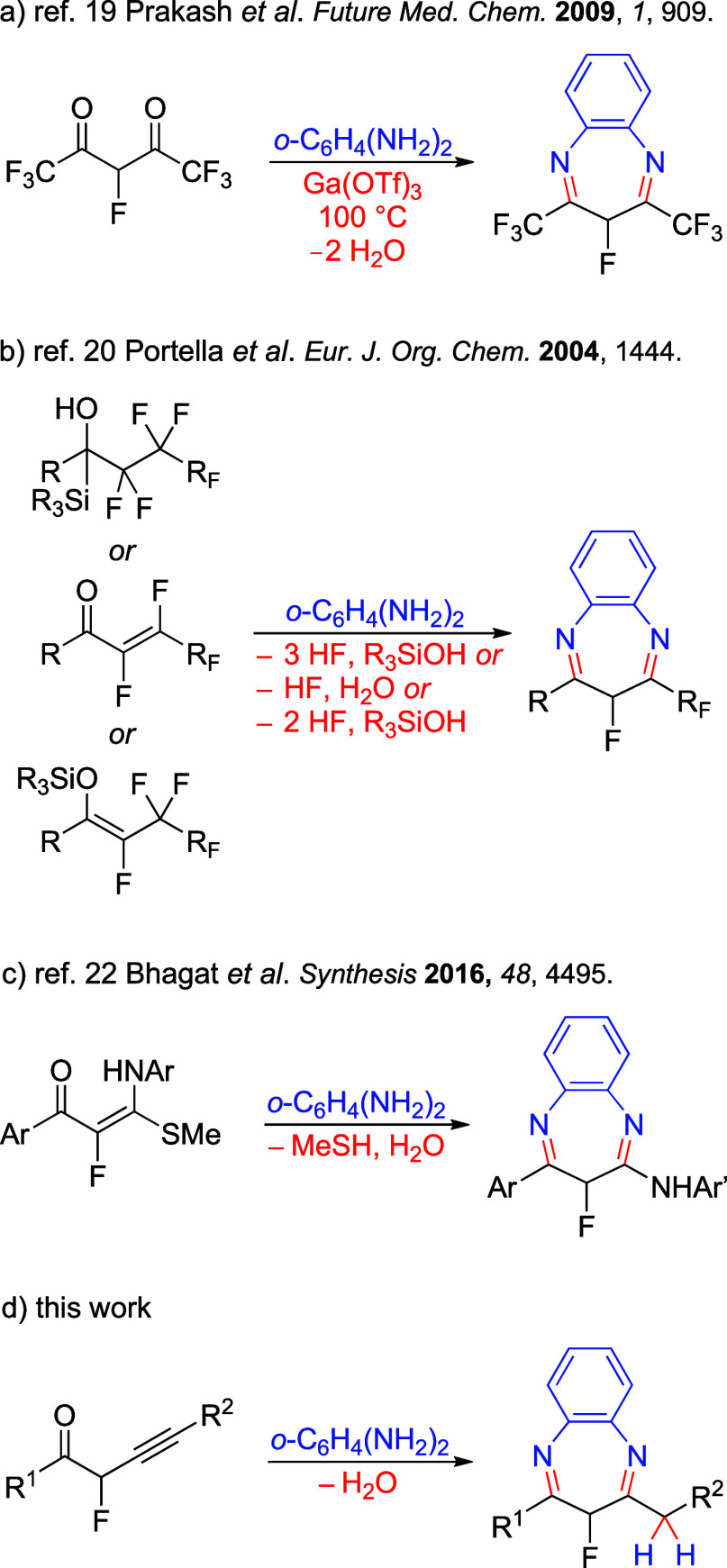
Representative methods for the synthesis of 3-*mono*fluoro-3*H*-benzo­[*b*]­[1,4]­diazepines.

## Results and Discussion

Alk-3-yn-1-ones (propargyl ketones, **1**, Scheme 1),^23^ containing a nonconjugated alkyne function, represent versatile bifunctional materials for reactions that lead to variously substituted furans,^24^ halofurans,^25^ pyrazoles,^26^ as well as nonfluorinated 1,5-benzodiazepines.^27,28^ Based on this reagent, we have already developed a convenient preparative fluorination protocol, superior to the earlier, much more cumbersome alternative synthetic routes.^29^ The alkynones **1** were converted to their *O*-*tert*-butyldimethylsilyl enol ethers (**2**). Subsequent fluorination reaction, using Selectfluor in acetonitrile, produced 2-fluoroalk-3-yn-1-ones (**3**), which were used as crude materials for further reactions.

**Scheme 1:**
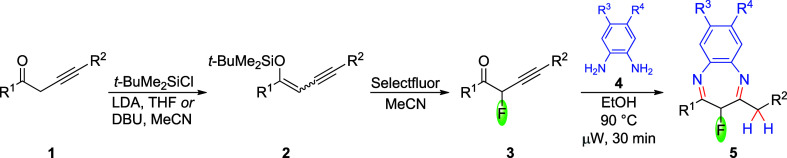
Preparation of 1,5-Benzodiazepines **5** from Alkynones **1**

The formation of C–N or C=N bonds can be achieved from alkynes via the hydroamination reaction that offers an atom-efficient pathway. Isolated alkynes typically require harsh conditions and the presence of a catalyst.^30^ Nevertheless, the reaction of fluoroalkynones **3** with equimolar amounts of symmetrical *o*-phenylenediamines (**4**) (ethanol, 90 °C) produced benzodiazepines (**5**) in high yields. The process, proceeding in reagent-grade ethanol and in the absence of a catalyst, was accelerated by microwave heating (Scheme 1). Compounds **5** were isolated by crystallization or column chromatography in 52–81% yields (Table 1).

**Table 1:** Preparation of Fluorinated 1,5-Benzodiazepines **5** from Fluoroalkynones **3**

entry	ynone	R^1^	R^2^	diamine	R^3^	R^4^	diazepine	yield [%]
1	**3a**	Ph	*p*-MeC_6_H_4_	**4a**	H	H	**5aa**	81
2	**3a**	Ph	*p*-MeC_6_H_4_	**4b**	Me	Me	**5ab**	75
3	**3a**	Ph	*p*-MeC_6_H_4_	**4c**	OMe	OMe	**5ac**	80
4	**3a**	Ph	*p*-MeC_6_H_4_	**4d**	(CH)_4_		**5ad**	68
5	**3b**	*p*-BrC_6_H_4_	*p*-MeC_6_H_4_	**4a**	H	H	**5ba**	72
6	**3b**	*p*-BrC_6_H_4_	*p*-MeC_6_H_4_	**4b**	Me	Me	**5bb**	69
7	**3c**	Ph	*c*-Pr	**4a**	H	H	**5ca**	65
8	**3c**	Ph	*c*-Pr	**4b**	Me	Me	**5cb**	68
9	**3c**	Ph	*c*-Pr	**4d**	(CH)_4_		**5cd**	63
10	**3d**	*p*-ClC_6_H_4_	*p*-*t*-BuC_6_H_4_	**4b**	Me	Me	**5db**	65

In addition to *o*-phenylenediamine (**4a**), 4,5-dimethyl- (**4b**) and 4,5-dimethoxy- (**4c**) derivatives, as well as 2,3-naphthalenediamine (2,3-diaminonaphthalene, **4d**) were included. The auxiliary groups R^1^ include phenyl and *p*-halophenyl substituents, whereas the corresponding R^2^ groups were *p*-tolyl and *p*-*tert*-butylphenyl, respectively. Using aryldiamines **4a**–**d** led to the formation of aryl/benzyl-substituted benzodiazepines **5aa**–**bb** (Table 1, entries 1–6), which were obtained in yields of 68–81%. Next, the scope of the reaction was extended to alkyl substituents as R^2^, introducing the cyclopropylmethylene group. Diazepines **5ca**,**cb**,**cd** containing phenyl R^1^ substituent and cyclopropylmethylene group were obtained using the same methodology with 65–69% yields, respectively (entries 7–9). *p*-Chlorophenyl/*p*-*tert*-butylbenzyl compound **5db** was prepared and isolated in 65% yield (entry 10).

NMR and HRMS confirmed the new benzodiazepine **5** structures. The characteristic ^1^H NMR features included a broad H–3 doublet (C_6_D_6_, 4.72–4.28 ppm, ^2^ *J*_HF_ = 43.2–51.6 Hz). The C*H*_2_C_3_H_5_ (methylene-cyclopropyl) diastereotopic signals of benzodiazepines **5ca**,**cb**,**cd** surfaced as separate ddd with an AB type intensity pattern (C_6_D_6_, 2.54 and 2.48–2.44 ppm with ^2^ *J*_HH_ = 14.6–15.7 Hz, ^3^ *J*_HH_ = 7.8–7.9 Hz, and ^4^ *J*_HF_ = 3.2–3.3 or 2.5–2.9 Hz, respectively, see the Supporting Information for spectra with expansions). In the ^13^C­{^1^H} NMR (C_6_D_6_) spectra, imine C=N signals appeared in the range of 165.8–155.4 ppm (^2^ *J*_CF_ = 20.5–23.7 Hz) and 159.0–149.3 (^2^ *J*_CF_ = 16.1–23.1 Hz). The ^19^F­{^1^H} NMR spectra displayed broad singlets in the range of 198.3–195.8 ppm. Intense molecular ions and accurate [M + H]^+^ peaks were observed in the HRMS spectra for compounds **5**. In addition to NMR data, X-ray crystallography unambiguously confirmed the predominant diimine (3*H*) tautomeric form of benzodiazepine **5aa** (*vide infra*), commonly observed for nonfluorinated benzodiazepines.^31^

### Crystallography

To gain better structural insight into 3-fluoro-1,5-benzodiazepines, crystallographic analysis was utilized to confirm the structure of the *p*-methylbenzyl-phenyl substituted benzodiazepine (**5aa**, Figure 3).^32^ A CCDC search of this chemical framework indicated a lack of outcomes for 3-*mono*fluorobenzodiazepines, apart from the reported single 2-arylamino derivative.^22^ Efforts to obtain diffraction-quality crystals were successful for compound **5aa**, using recrystallization from ethanol. The fluorobenzodiazepine **5aa** exists in the solid state as the 3*H* tautomeric form with two imino bonds, as indicated by N1–C2 1.2880(19) Å and N5–C4 1.282(2) Å bond distances. Analysis of the crystal structure revealed a half-boat conformation of the seven-membered ring characterized by a folding angle of 90.0°. The geometry of the 7-membered ring of **5aa** is alternatively described by determining the ring flip angle, defined as the distance *d* of the distal carbon atom (C–3) to the plane containing the benzene ring (nitrogen atoms not included).^33^ For **5aa**, the *d* structural parameter was 1.44 Å, relatively similar to the nonfluorinated analog with the same substituents (*d* = 1.41 Å; CCDC 929588). Comparably, the calculated *d* values for related nonfluorinated 1,5-benzodiazepines range between 1.31 and 1.52 Å (average of 1.46 Å).^34^ The fluorine atom always occupied an equatorial position in the solid state in all crystallographically independent molecules (enantiomers), which is illustrated in the packing diagram (Supporting Information Figure S1). The geometry of a single molecule is stabilized primarily by π-stacking interactions between the phenyl and 4-methylphenyl rings (centroid distance of 3.740 Å, shift distance of 1.194 Å, angle of 6.836°). An additional stabilizing factor includes the F1···H17–C17 intramolecular hydrogen bond (D–A length 2.822 Å).

**Figure 3:**
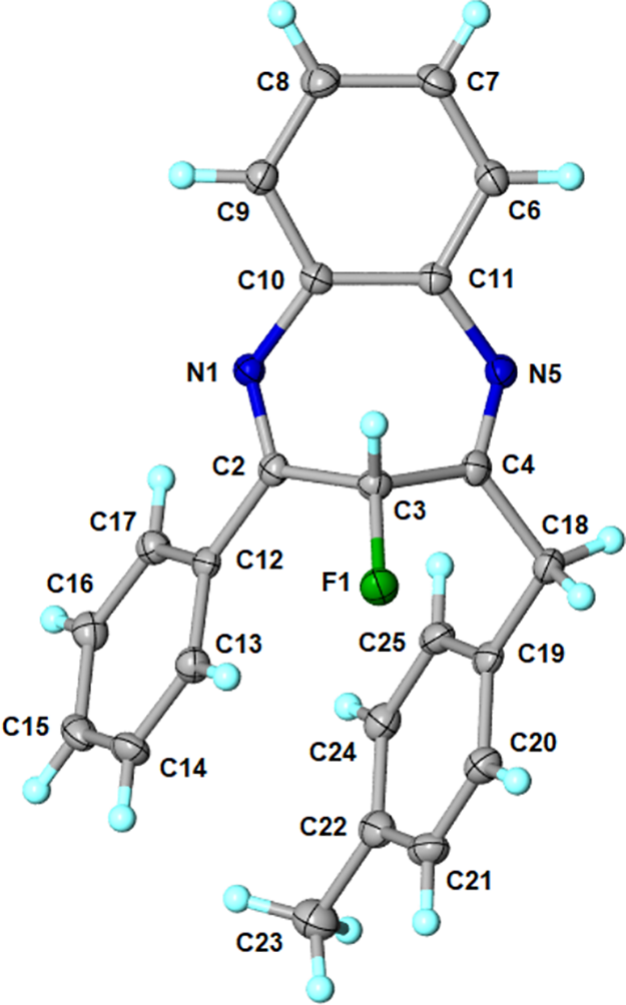
Thermal ellipsoids view of fluorobenzodiazepine **5aa** at the 50% probability level with the crystallographic atom-labeling scheme. Selected interatomic distances (Å): F1–C3 1.3901(16), N1–C2 1.2880(19), N1–C10 1.4090(19), C2–C3 1.523(2), C3–C4 1.512(2), N5–C4 1.282(2), C4–C18, 1.510(2), N5–C11 1.4118(19), C10–C11 1.418(2). Key angles (°): F1–C3–C2 114.36(12), F1–C3–C4 110.93(12), C2–N1–C10, 122.27(13), N1–C2–C3 118.45(13), C2–C3–C4 106.81(12), C3–C4–N5 120.40(13), C4–N5–C11 120.95(13), N1–C10–C11 124.48(13), N5–C11–C10 125.25(13).

### NMR Conformational Studies

The conformational interconversion of a half-boat conformation levels out the nonequivalence of the two C–3 methylene protons in solution at certain temperatures for nonfluorinated 1,5-diazepines.^28^ Although ring inversion in six-membered structures such as fluorocyclohexanes is well established,^35^ to our knowledge, fluorobenzodiazepines have not been investigated by ^19^F NMR. To evaluate the conformation of the fluorinated seven-membered ring, C–3 substituents were tracked by NMR variable temperature studies. ^1^H and ^19^F NMR spectra of **5aa** were recorded at temperatures ranging from 194 to 353 K (−79 to +80 °C; 400 MHz, CDCl_3_; Figure 4a). At room temperature (293 K), the ^1^H signal of the proton geminal to fluorine tends to disappear into the baseline; however, when the spectrum is magnified, a broad doublet at 4.41 ppm (in CDCl_3_) with *J*_HF_ = 43.2 Hz is noticeable. Decreasing the sample temperature creates a more distinct signal positioned further upfield. The slower ring inversion allows for signal resolution of the C–3 proton in equatorial and axial conformations with approximately comparable ratios; the overlap with the peaks attributed to the benzyl diastereotopic protons rendered integration ambiguous. When the temperature was increased, the signal once again became clearer, shifting downfield. The benzylic CH_2_ signal at around 3.8 ppm displayed a negligible change in chemical shift as the temperature was adjusted. As the temperature decreased, the larger effect of proton differentiation is likely due to slower rotation, which was observed, and the analysis of ^19^F signals in the same experiment shows related trends (Figure 4b). At 293 K, the ^19^F signal of the methine fluorine was hard to detect and appeared as a broad singlet at −196.1 ppm; a change in temperature results in a downfield shift and noticeable splitting, as the signals appear as doublets with *J*_HF_ = 44.4 Hz. These findings suggest that indeed the conformational interconversion exists in the case of **5aa**, and that at room temperature there is an equilibrium between the two conformers, while at lower temperatures, one is slightly more stable and predominates. Considering various effects that can influence equatorial and axial conformational preferences,^36^ due to the absence of in-depth specialized studies,^37^ we refrained from presenting quantitative conclusions on stability.

**Figure 4:**
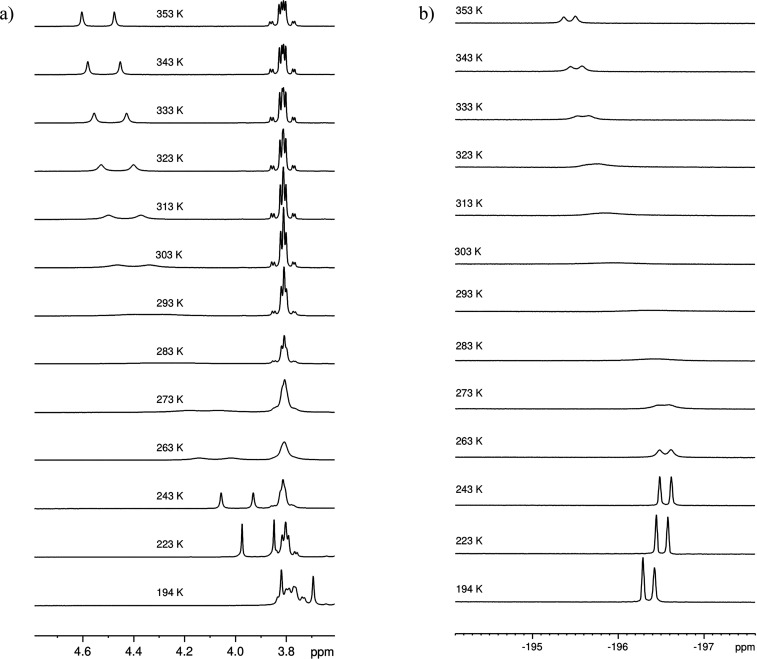
Variable temperature NMR (CDCl_3_) study for **5aa**: (a) ^1^H NMR (400 MHz) and (b) ^19^F NMR.

### Computational Mechanistic Elucidation

The reaction mechanism outline is discussed based on the presumption that the alkyne moiety serves as an initial site of reactivity of 2-fluoroalk-3-yn-1-one **3**, which, as determined by Density Functional Theory (DFT) calculations, exists predominantly in a keto form (the enol form is +9.3 kcal/mol less stable). The process begins via tautomerization of nonconjugated **3** to allenone **6** (Scheme 2), which is marginally more stable than the corresponding alkyne **3** (−1.2 kcal/mol). This creates an opportunity for the aza-Michael conjugate addition, which forms enamine structure **7** (*E*/*Z* isomers can be considered), formally a product of hydroamination of alkynone **3**. Subsequent migration of the double bond leads to conjugated enaminone **8**. A six-membered ring intramolecular hydrogen bonding interaction enforces the stereospecificity of the isomerization, promoting formation of the *E* form (pathway A, Scheme 2). Subsequent cyclization to form benzodiazepine **9**, and its tautomerization, lead to the final product **5**. Pathway A, leading to isolable enaminone **8** (*vide infra*), may be favored due to the migration of the C=C double bond into conjugation with the carbonyl group. The isolated enamine structure **7** may undergo cyclization to give benzodiazepine **10**, whose tautomerization leads to benzodiazepine **5** (pathway B, Scheme 2). Alternatively, the tautomerization of **7** into its imino form **11**, followed by cyclization, cannot be excluded (pathway C, Scheme 2).

**Scheme 2:**
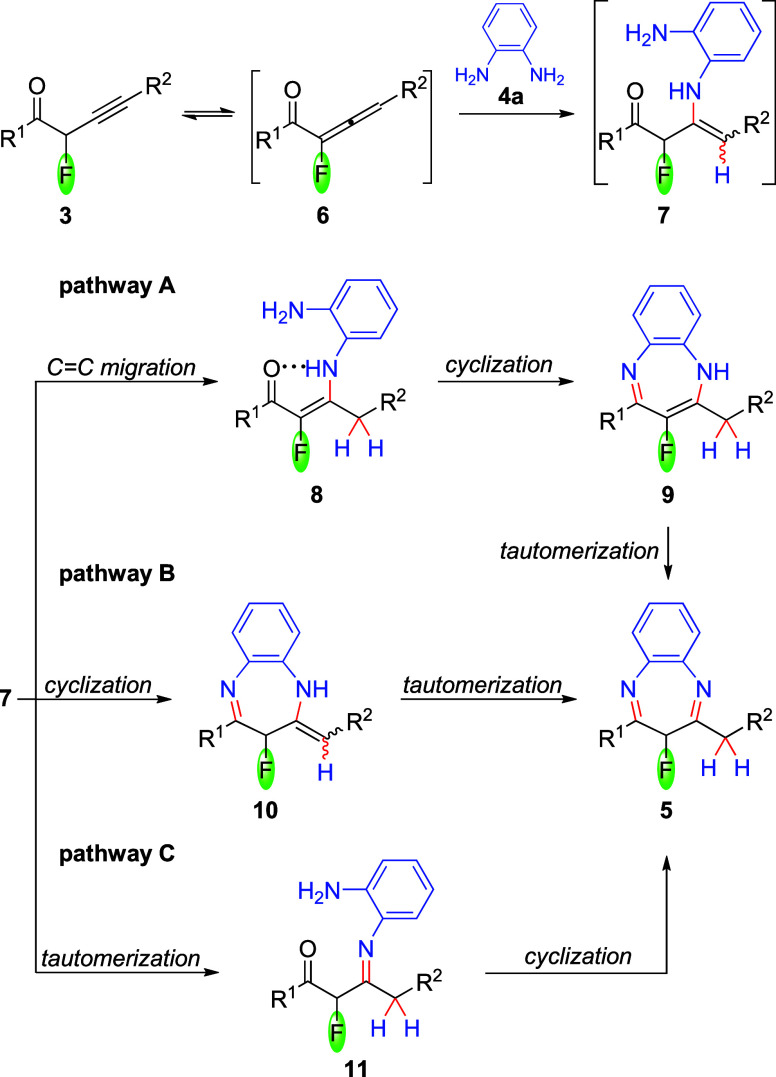
Plausible Mechanistic Pathways for the Synthesis of Benzodiazepines **5**

To further develop mechanistic insight and suggest potential pathways, extensive electronic-structure modeling of the plausible intermediates and transition states (TS) involved in the pathways illustrated in Scheme 2 was carried out. The resulting energy vs reaction coordinate profiles along pathways A, B, and C are visualized in Figure 5, using the optimized structures and their Gibbs energies *G* at 1 atm and 298 K calculated for all species at the ωB97X-D/aug-pcseg-1/SMD (ethanol) level of theory, as described in detail in the Experimental Section. All Gibbs energies are given relative to the *G* value *G*(**7**) for enaminone **7**, Δ*G* = *G*-*G*(**7**). In Figure 5, we use compound labels (**3**, **5**–**10**) identical to those in general Scheme 2 to maintain internal consistency of this section and avoid the overcrowding of Figure 5. However, actual calculations have been performed for the exact structures shown in Figure 5, most relevant for this study. The raw data are available in the Supporting Information (SI).

**Figure 5:**
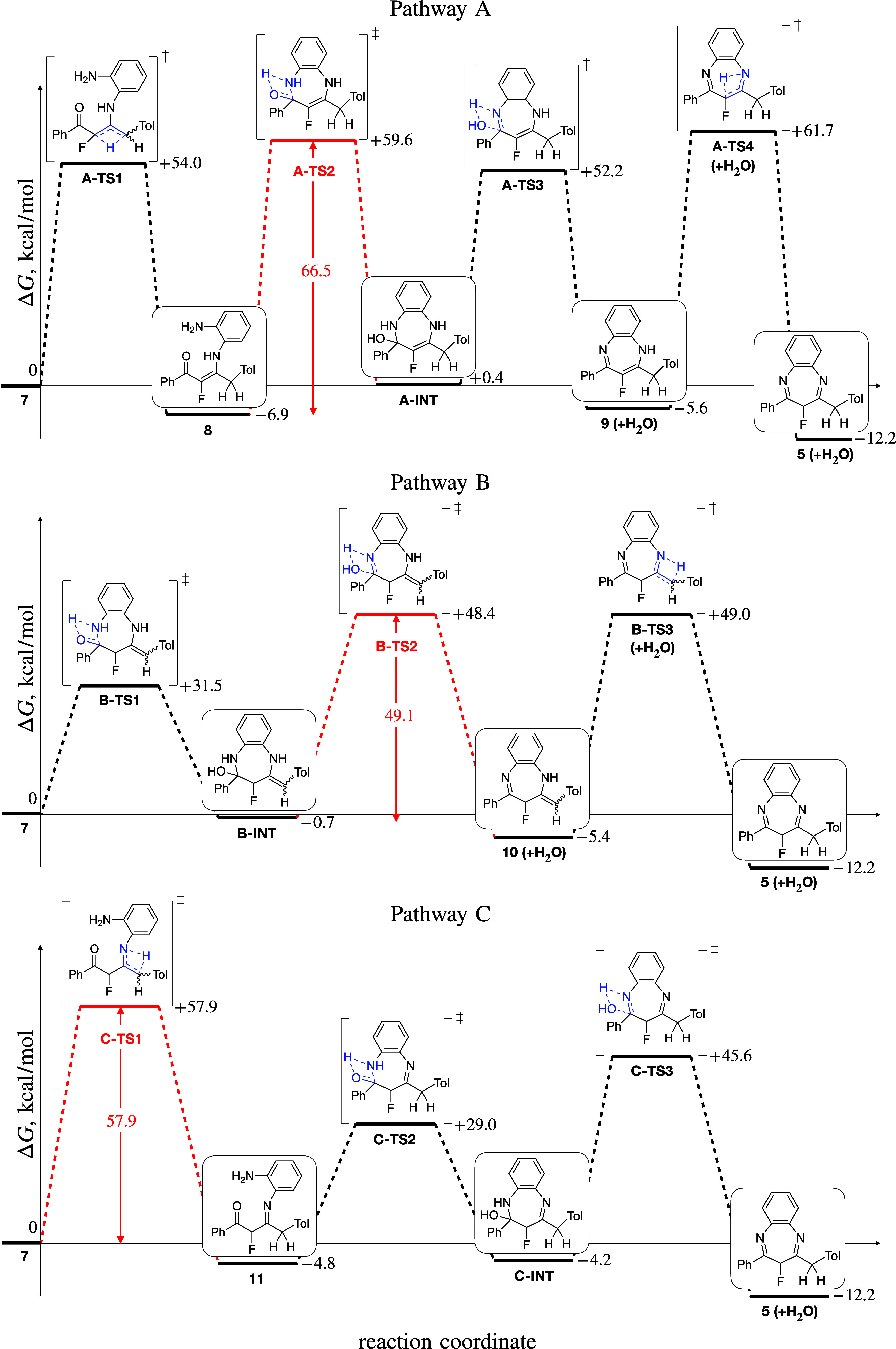
Calculated Gibbs energies *G* at 1 atm (101.325 kPa) and 298 K for pathways A (top), B (center), and C (bottom) for corresponding mechanistic pathways reactions coordinate, starting from enaminone **7** (c.f. Scheme 2), for the synthesis of benzodiazepine **5aa**.

To discern among pathways A–C, the rate-determining step using the activation Gibbs energy was identified. For each pathway, this activation energy Δ_max_ *G* ^‡^ was calculated as the maximum difference between a transition state and the preceding reactant or intermediate. Given that steps involve dehydration, the comparability of the Gibbs energies was maintained by imposing the material balance condition. To this end, the Gibbs energy of an isolated water molecule at its equilibrium geometry was calculated at the same level of theory as for all other species and added to the Gibbs energy of the product of dehydration. In the calculations for Figure 5, a designation (+H_2_O) is added to all structures where it is necessary to maintain a material balance.

For the three pathways, the evaluation of the maximum Gibbs energy differences, or activation Gibbs energies Δ_max_ *G* ^‡^ as defined above, gives the following results (Figure 5). For pathway A, Δ_max_ *G* ^‡^(A) = *G*(**A-TS4**)–*G*(**9**) = 67.3 kcal/mol is formally the largest, although Δ_max_ *G* ^‡^(A) = *G*(**A-TS2**)–*G*(**8**) = 66.5 kcal/mol is lower by less than 1 kcal/mol and thus virtually indiscernible from the former value, making the cyclization of enaminone **8** via **A-TS2** to form amino alcohol **A-INT** the actual rate-determining step. For pathways B and C, the activation Gibbs energies are Δ_max_ *G* ^‡^(B) = *G*(**B-TS3**)–*G*(**9**) = 54.4 kcal/mol and Δ_max_ *G* ^‡^(C) = *G*(**C-TS1**)–*G*(**7**) = 57.9 kcal/mol, respectively. Therefore, pathway B features the fastest rate-determining step, which is the dehydration of **B-INT** into **10** via **B-TS2**. Additionally, the initial cyclization step along pathway B requires an activation energy Δ_1_ *G* ^‡^(B) = *G*(**B-TS1**)–*G*(**7**) = 31.5 kcal/mol, which is significantly lower than the activation energies of initial tautomerizations Δ_1_ *G* ^‡^(A) = *G*(**A-TS1**)–*G*(**7**) = 54.0 kcal/mol and Δ_1_ *G* ^‡^(C) = *G*(**C-TS1**)–*G*(**7**) = 57.9 kcal/mol. As a result, pathway B appears strongly kinetically favored, indicating that it is the most likely overall. At the same time, the high kinetic barriers of all steps make pathway A the least plausible.

Alkynones **1** react with *o*-phenylenediamines **4** to produce nonfluorinated enaminones. However, reported compounds containing a fluoro-enaminone scaffold are observed as a combination of the enamino and imino forms.^38^ We were intrigued to isolate a plausible intermediate illustrated on Scheme 2, such as **7** vs **8**, and at the same time to investigate a divergent reaction of fluoroalkynones **3** with *o*-phenylenediamines. The alkynone **3a** was combined with equimolar amounts of *o*-phenylenediamine **4a** (Scheme 3). In order to prevent the formation of cyclization products, which were initially noticed in the post-reaction mixtures, triethylamine was added. The reaction was carried out at room temperature in ethanol/triethylamine (22 °C, 99:1 v/v) and led to the formation of conjugated (more stable) fluoro-enaminone **8aa**, characterized by NMR in its crude form. The characteristic ^1^H NMR signals for enaminone **8aa** (C_6_D_6_) included the deshielded NH protons (11.73 ppm, due to intramolecular hydrogen bonding) and a free amino NH_2_ group (3.00 ppm). The GC/MS spectrum exhibited an *m*/*z* base peak of [M–H_2_O]^+^, resulting from a dehydration reaction during acquisition.

**Scheme 3:**
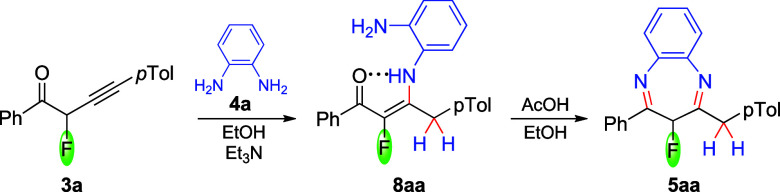
Reaction Leading to Enaminone **8aa**, and Its Cyclization

We confirmed experimentally that conjugated enaminone **8aa** requires acid catalysis (acetic acid) to effect the cyclocondensation reaction, yielding benzodiazepine **5aa**, according to pathway A (Scheme 3). This result is in agreement with computational assessment that pathway B (Scheme 2, Figure 5) is the most likely mechanistic contributor since it is more difficult to advance from enaminone **8aa**, as compared to the direct formation of benzodiazepine from fluoroalkynone **3** (via enaminone **7**; pathway B).

## Conclusions

In summary, we presented a novel and attractive protocol that provides a convenient method for the preparation of monofluorinated benzodiazepines. We have brought 2-fluoro-alk-3-yn-1-one, a bifunctional reagent that contains a nonconjugated alkyne moiety, into reactions of the regiospecific amination of the alkyne group, leading to diversely substituted 3-fluoro-1,5-benzodiazepines. This synthetic approach utilizes mild conditions, eliminates the need for a catalyst, and employs reagent-grade ethanol, a renewable solvent. One-pot establishment of two C=N and two C–H bonds with high atom economy allowed for the introduction of substituents such as benzyl or cyclopropylmethylene at the C–2 position of the diazepine. Variable temperature NMR studies indicate the presence of comparable numbers of axial and equatorial conformers. The computational mechanistic studies identified a plausible pathway that forms a cyclic structure followed by its tautomerization to the final benzodiazepine form.

## Experimental Section

### General Methods

The microwave reactions were carried out using capped vials in a Biotage Initiator reactor equipped with a conventional temperature IR sensor and stirring capabilities. NMR spectra were obtained with a Bruker Avance III spectrometer (^1^H of 400 MHz, ^13^C of 100 MHz, and ^19^F of 376 MHz). The chemical shifts were reported in δ (ppm) values relative to C_6_D_6_, CDCl_3,_ and (external) CF_3_COOH (7.16/128.06, 7.26/77.16, and 0 ppm, respectively). Mass spectra were recorded on an Agilent 6520 Q-TOF LCMS (HRMS) and Hewlett-Packard 5973 GC/MS instruments. IR spectra were recorded on a Bruker Alpha-P ATR spectrometer. Melting points (closed capillaries) were recorded on a Mel Temp apparatus. All products were stored in a refrigerator (4 °C), except fluoroalkynones **3** that were stored in the freezer (−80 °C). 4-(4′-Methylphenyl)-1-phenylbut-3-yn-1-one **1a**,^22^ 2-fluoro-4-(4′-methylphenyl)-1-phenylbut-3-yn-1-one **3a**,^29^ and 4-cyclopropyl-2-fluoro-1-phenylbut-3-yn-1-one **3c** ^29^ were prepared according to literature procedures.

#### 1-(4′-Bromophenyl)-2-fluoro-4-(4”-methylphenyl)-but-3-yn-1-one (**3b**)

The compound **3b** was prepared according to the method described,^29^ and was obtained as a crude yellowish solid quantitatively, mp 85–86 °C. *R*_f_ 0.75 (hexanes/ethyl acetate 4:1 v/v, 254 nm). MS (ESI, *m*/*z*): 333 ([M­{^81^Br} + H]^+^, 100%), 331 ([M­{^79^Br} + H]^+^, 100%). HRMS (ESI-TOF) [M + H]^+^ calcd for C_17_H_13_BrFO 331.0134, found 331.0131. IR (cm^–1^, ATR): 2216 w (C≡C), 1711 vs (C=O), 1062 vs, 1006 s, 935 vs, 814 vs, 618 m (C–Br). ^1^H NMR (CDCl_3_, 400 MHz): δ 8.01 (AA′XX′, 2H, *J*_HH_ = 8.4 Hz), 7.65 (AA′XX′, 2H, *J*_HH_ = 8.6 Hz), 7.31 (AA′XX′, 2H, *J*_HH_ = 8.0 Hz), 7.12 (AA′XX′, 2H, *J*_HH_ = 8.0 Hz), 6.18 (d, 1H, *J*_HF_ = 49.1 Hz), 2.34 (s, 3H). ^13^C­{^1^H} NMR (CDCl_3_, 100 MHz): δ 189.0 (d, *J*_CF_ = 22.3 Hz), 140.3, 132.2, 132.0 (d, *J*_CF_ = 2.9 Hz), 131.5 (d, *J*_CF_ = 1.3 Hz), 131.0 (d, *J*_CF_ = 2.2 Hz), 129.7, 129.3, 117.7 (d, *J*_CF_ = 4.0 Hz), 93.6 (d, *J*_CF_ = 10.9 Hz), 83.6 (d, *J*_CF_ = 185.0 Hz), 80.1 (d, *J*_CF_ = 26.0 Hz), 21.6. ^19^F­{^1^H} NMR (C_6_D_6_, 376 MHz): δ −177.8 (s).

#### Synthesis of 3-Fluoro-1,5-benzodiazepines **5**, General Procedure

A 10 mL round-bottom microwave vial equipped with a stir bar was charged with fluoroalkynone **3** (0.50 mmol), diamine **4** (0.55 mmol), and ethanol (3 mL). The vial was sealed, and the reaction mixture was irradiated in the microwave reactor at 90 °C for 30 min with stirring. The solvent was removed by rotary evaporation.

Purification of compounds **5aa**, **5ba**, and **5bb**: The vial was allowed to reach rt, and the solvent was removed by rotary evaporation and oil pump vacuum. The residue was recrystallized from ethanol (0.5 mL) at 4 °C overnight. The solid was filtered off, washed with cold hexanes (2 × 0.5 mL), and dried under an oil pump vacuum to give the product. The filtrate was evaporated and subjected to column chromatography (silica gel, hexanes/ethyl acetate 30:1 v/v) to give an additional amount of the product.

Purification of compounds **5ab**, **5ca**, **5cb**, **5ac**, and **5cd**: The vial was allowed to cool to rt and the solvent was removed by rotary evaporation and oil pump vacuum. The residue was filtered through silica gel plug (∼10 cm, hexanes/ethyl acetate 80:20 v/v). TLC under 254 nm displayed a purple spot for the final product, while 365 nm wavelength displayed noncharacterized decomposition products. Silica gel column chromatography (15 × 2.5 cm; hexanes/ethyl acetate 95:5 v/v) gave a yellow fraction. The solvent was removed by rotary evaporation, and the residue was dried under oil pump vacuum to yield the pure product as a yellow oil or wax.

#### 3-Fluoro-4-(4-methylbenzyl)-2-phenyl-3*H*-benzo­[*b*]­[1,4]­diazepine (**5aa**)

From **3a** (0.157 g, 0.622 mmol) and *o*-phenylenediamine **4a** (0.0710 g, 0.657 mmol), light yellow crystals of **5aa** (total yield of 0.173 g, 0.505 mmol, 81%), mp 71–72 °C. HRMS (ESI-TOF) [M + H]^+^ calcd for C_23_H_20_FN_2_ 343.1611, found 343.1601. IR (cm^–1^, ATR): 1628 w (C=N), 1564 w (C=N), 1236 m, 1099 s, 758 vs. ^1^H NMR (C_6_D_6_, 400 MHz): δ 7.69 (d, 2H, ^3^ *J*_HH_ = 8.0 Hz), 7.66–7.55 (m, 2H), 7.09–6.98 (m, 5H), 6.96 (d, 2H, ^3^ *J*_HH_ = 7.8 Hz), 6.70 (d, 2H, ^3^ *J*_HH_ = 7.8 Hz), 4.41 (bd, 1H, ^2^ *J*_HF_ = 43.2 Hz), 3.86 (dd, 2H, ^2^ *J*_HH_ = 14.1 Hz, ^4^ *J*_HF_ = 4.1 Hz), 3.81 (dd, 2H, ^2^ *J*_HH_ = 14.1 Hz, ^4^ *J*_HF_ = 3.2 Hz), 1.98 (s, 3H). ^13^C­{^1^H} NMR (C_6_D_6_, 100 MHz): δ 159.8 (d, ^2^ *J*_CF_ = 23.7 Hz), 153.9 (d, ^2^ *J*_CF_ = 16.1 Hz), 140.4, 139.9, 136.2, 135.1 (d, ^3^ *J*_CF_ = 3.0 Hz), 132.7, 130.2, 130.1, 129.7, 129.3, 128.9, 128.4, 126.1, 126.0, 91.4 (d, ^1^ *J*_CF_ = 194.5 Hz), 40.9, 21.0. ^19^F­{^1^H} NMR (C_6_D_6_, 376 MHz): δ −196.1 (br s).

#### 3-Fluoro-8,9-dimethyl-4-(4-methylbenzyl)-2-phenyl-3*H*-benzo­[*b*]­[1,4]­diazepine (**5ab**)

From **3a** (0.140 g, 0.555 mmol) and 4,5-dimethyl-1,2-phenylenediamine **4b** (0.0810 g, 0.595 mmol), a light yellow dense oil of **5ab** (total yield of 0.154 g, 0.416 mmol, 75%). HRMS (ESI-TOF) [M + H]^+^ calcd for C_25_H_24_FN_2_ 371.1924, found 371.1926. IR (cm^–1^, ATR): 1612 w (C=N), 1568 w (C=N), 1262 m, 1103 vs, 759 s. ^1^H NMR (C_6_D_6_, 400 MHz): δ 7.83–7.73 (m, 2H), 7.45 (s, 2H), 7.12–6.98 (m, 5H), 6.74 (d, 2H, ^3^ *J*_HH_ = 7.8 Hz), 4.66 (bd, 1H, ^2^ *J*_HF_ = 49.7 Hz), 3.93 (dd, 2H, ^2^ *J*_HH_ = 14.2 Hz, ^4^ *J*_HF_ = 4.0 Hz), 3.85 (dd, 2H, ^2^ *J*_HH_ = 14.2 Hz, ^4^ *J*_HF_ = 3.0 Hz), 2.04 (s, 6H), 2.00 (s, 3H). ^13^C­{^1^H} NMR (C_6_D_6_, 100 MHz): δ 158.3 (d, ^2^ *J*_CF_ = 20.6 Hz), 152.4 (d, ^2^ *J*_CF_ = 19.0 Hz), 138.5, 138.1, 136.1, 135.4 (d, ^3^ *J*_CF_ = 2.9 Hz), 135.2, 135.0, 133.0, 130.1, 130.0, 129.8, 129.3, 128.8, 91.4 (bd, ^1^ *J*_CF_ = 197.3 Hz), 40.9, 21.0, 19.4­(2C). ^19^F­{^1^H} NMR (C_6_D_6_, 376 MHz): δ −195.8 (br s).

#### 3-Fluoro-8,9-dimethoxy-4-(4-methylbenzyl)-2-phenyl-3*H*-benzo­[*b*]­[1,4]­diazepine (**5ac**)

From **3a** (0.3040 g, 1.205 mmol) and 4,5-dimethoxybenxene-1,2-diamine **4c** (0.2130 g, 1.266 mmol). Silica gel column chromatography (15 × 2.5 cm; hexanes/ethyl acetate 95:5 to 80:20 v/v) gave a yellow wax of **5ac** (0.388 g, 0.965 mmol, 80%). *R*_f_ = 0.52 (hexanes/ethyl acetate 5:3). HRMS (ESI-TOF) [M + H]^+^ calcd for C_25_H_24_FN_2_O_2_ 403.1816, found 403.1822. IR (cm^–1^, FTIR): 1611 m (C=N), 1502 s (C=N), 1264 s, 1102 s. ^1^H NMR (C_6_D_6_, 400 MHz): δ 7.93–7.79 (m, 2H), 7.10–7.07 (m, 3H), 7.07–7.05 (m, 2H), 7.05–7.01 (m, 2H), 6.78 (s, 1H), 6.76 (s, 1H), 4.68 (bd, 1H, ^2^ *J*_HF_ = 48.5 Hz), 3.97 (dd, 1H, ^2^ *J*_HH_ = 14.5 Hz, ^4^ *J*_HF_ = 3.8 Hz), 3.86 (dd, 1H, ^2^ *J*_HH_ = 14.5 Hz, ^4^ *J*_HF_ = 2.6 Hz), 3.40 (s, 3H), 3.36 (s, 3H), 2.00 (s, 3H). ^13^C­{^1^H} NMR (C_6_D_6_, 100 MHz): δ 155.4 (d, ^2^ *J*_CF_ = 21.9), 149.3 (d, ^2^ *J*_CF_ = 21.2 Hz), 149.0, 148.8, 136.2, 135.5 (d, ^3^ *J*_CF_ = 2.9 Hz), 134.7, 134.4, 133.2, 130.1, 130.05, 129.98, 129.8, 129.4, 110.3, 109.8, 91.9 (bd, ^1^ *J*_CF_ = 197.1 Hz), 55.5, 55.4, 40.7, 21.0. ^19^F­{^1^H} NMR (C_6_D_6_, 376 MHz): δ −196.5 (br s).

#### 3-Fluoro-4-(4-methylbenzyl)-2-phenyl-3*H*-naphtho­[*b*]­[1,4]­diazepine (**5ad**)

From **3a** (0.128 g, 0.506 mmol) and 2,3-diaminonaphthalene **4d** (0.0840 g, 0.531 mmol), a yellow wax of **5ad** (overall yield of 0.135 g, 0.344 mmol, 68%). *R*_f_ = 0.5 (hexanes/ethyl acetate 6:1). HRMS (ESI-TOF) [M + H]^+^ calcd for C_27_H_22_FN_2_ 393.1762, found 393.1763. IR (cm^–1^, FTIR): 1639 (C=N), 1604 (C=N), 1288 s, 1105 s. ^1^H NMR (C_6_D_6_, 400 MHz): δ 8.10 (d, 2H, ^4^ *J*_HH_ = 1.3 Hz), 7.76 (dt, 2H, ^3^ *J*_HH_ = 8.2 Hz, ^4^ *J*_HH_ = 1.5 Hz), 7.65 (dd, 2H, ^3^ *J*_HH_ = 6.2 Hz), 7.21 (dd, 2H, ^3^ *J*_HH_ = 6.3 Hz), 7.11–7.02 (m, 5H), 6.77 (s, 1H), 6.75 (s, 1H), 4.72 (bd, 1H, ^2^ *J*_HF_ = 50.7 Hz), 3.96 (ddd, 1H, ^2^ *J*_HH_ = 14.2 Hz, ^4^ *J*_HF_ = 4.1 Hz), 3.89 (ddd, 1H, ^2^ *J*_HH_ = 14.2 Hz, ^4^ *J*_HF_ = 3.4 Hz), 2.01 (s, 3H). ^13^C­{^1^H} NMR (C_6_D_6_, 100 MHz): δ 164.7 (d, ^2^ *J*_CF_ = 22.3 Hz), 159.0 (d, ^2^ *J*_CF_ = 19.7 Hz), 139.6, 139.1, 136.4, 135.6 (d, ^3^ *J*_CF_ = 2.6 Hz), 132.7, 132.0, 131.9, 130.3, 129.9, 129.8, 129.4, 128.7, 126.8, 126.5, 126.4, 126.2, 90.1 (bd, ^1^ *J*_CF_ = 199.6 Hz), 41.7, 21.0. ^19^F­{^1^H} NMR (C_6_D_6_, 376 MHz): δ −196.1­(br s).

#### 2-(4-Bromophenyl)-3-fluoro-4-(4-methylbenzyl)-3*H*-benzo­[*b*]­[1,4]­diazepine (**5ba**)

From **3b** (0.166 g, 0.500 mmol) and *o*-phenylenediamine **4a** (0.0540 g, 0.550 mmol). The solid was filtered off from the post-reaction mixture and washed with cold ethanol (5 × 0.5 mL), to give a dark yellow solid of **5ba** (0.133 g, 0.317 mmol, 63%). Silica gel column chromatography (15 × 2.5 cm; hexanes/ethyl acetate 30:1 v/v) gave additional **5ba** (0.0186 g, 0.0439 mmol, 9%). Overall yield (0.152 g, 0.361 mmol, 72%), mp 120–121 °C. HRMS (ESI-TOF) [M + H]^+^ calcd for C_23_H_19_BrFN_2_ 421.0716, found 421.0715. IR (cm^–1^, ATR): 1510 w (C=N), 1234 w, 1097 s, 765 vs (C–Br). ^1^H NMR (C_6_D_6_, 400 MHz): δ 7.67–7.52 (m, 2H), 7.26 (d, 2H, ^3^ *J*_HH_ = 7.8 Hz), 7.12–7.01 (m, 4H), 6.81 (AA′XX′, 2H, ^3^ *J*_HH_ = 7.8 Hz), 6.59 (d, 2H, ^3^ *J*_HH_ = 7.8 Hz), 4.28 (bd, 1H, ^2^ *J*_HF_ = 47.3 Hz), 3.81 (d, 2H, ^4^ *J*_HH_ = 4.0 Hz), 2.00 (s, 3H). ^13^C­{^1^H} NMR (C_6_D_6_, 100 MHz): δ 159.9 (d, ^2^ *J*_CF_ = 20.5 Hz), 151.6 (d, ^2^ *J*_CF_ = 22.0 Hz), 140.2, 139.7, 136.6, 133.6 (d, ^3^ *J*_CF_ = 3.2 Hz), 132.1, 131.74, 131.67, 131.1, 129.7, 129.2, 128.9, 126.3, 126.1, 125.0, 91.4 (bd, ^1^ *J*_CF_ = 215.8 Hz), 41.2, 21.0. ^19^F­{^1^H} NMR (C_6_D_6_, 376 MHz): δ −196.6 (br s).

#### 2-(4-Bromophenyl)-3-fluoro-8,9-dimethyl-4-(4-methylbenzyl)-3*H*-benzo­[*b*]­[1,4]­diazepine (**5bb**)

From **3b** (0.166 g, 0.500 mmol) and 4,5-dimethyl-1,2-phenylenediamine **4b** (0.0680 g, 0.500 mmol). The solid was filtered off from the post-reaction mixture and washed with cold ethanol (4 × 0.5 mL), to give a dark yellow solid of **5bb** (0.128 g, 0.285 mmol, 57%). Silica gel column chromatography (15 × 2.5 cm; hexanes/ethyl acetate 30:1 v/v) gave additional **5bb** (0.0268 g, 0.0598 mmol, 12%). Overall yield (0.155 g, 0.345 mmol, 69%), mp 121–122 °C. HRMS (ESI-TOF) [M + H]^+^ calcd for C_25_H_23_BrFN_2_ 449.1029, found 449.1029. IR (cm^–1^, ATR): 1609 w (C=N), 1581 m (C=N), 1070 m, 1005 s, 886 vs, 754 s (C–Br). ^1^H NMR (C_6_D_6_, 400 MHz): δ 7.47 (s, 1H), 7.42 (s, 1H) 7.35 (d, 2H, ^3^ *J*_HH_ = 7.9 Hz), 7.12 (AA’XX’, 2H, ^2^ *J*_HH_ = 8.6 Hz), 6.88 (d, 2H, ^3^ *J*_HH_ = 7.8 Hz), 6.63 (d, 2H, ^3^ *J*_HH_ = 7.8 Hz), 4.53 (bd, 1H, ^2^ *J*_HF_ = 49.8 Hz), 3.88 (dd, 2H, ^2^ *J*_HH_ = 13.6 Hz, ^4^ *J*_HF_ = 3.9 Hz), 3.84 (dd, 2H, ^2^ *J*_HH_ = 13.6 Hz, ^4^ *J*_HF_ = 3.2 Hz), 2.04 (s, 6H), 2.02 (s, 3H). ^13^C­{^1^H} NMR (C_6_D_6_, 100 MHz): δ 158.5 (d, ^2^ *J*_CF_ = 23.5 Hz), 151.2 (d, ^2^ *J*_CF_ = 19.0 Hz), 138.4, 138.0, 136.5, 135.5, 135.2, 133.9 (d, ^3^ *J*_CF_ = 3.1 Hz), 132.4, 131.7, 131.6, 131.1, 129.7, 129.3, 128.8, 124.9, 91.4 (bd, ^1^ *J*_CF_ = 206.4 Hz), 41.2, 21.0, 19.40, 19.36. ^19^F­{^1^H} NMR (C_6_D_6_, 376 MHz): δ −196.2 (br s).

#### 4-(Cyclopropylmethyl)-3-fluoro-2-phenyl-3*H*-benzo­[*b*]­[1,4]­diazepine (**5ca**)

From **3c** (0.139 g, 0.687 mmol) and *o*-phenylenediamine **4a** (0.0800g, 0.740 mmol), a yellow oil of **5ca** (0.131 g, 0.447 mmol, 65%). *R*_f_ = 0.45 (hexanes/ethyl acetate 5:1). HRMS (ESI-TOF) [M + H]^+^ calcd for C_19_H_17_FN_2_ 293.1449, found 293.1461. IR (cm^–1^, FTIR): 1629 (C=N), 1607 (C=N), 1104 s. ^1^H NMR (C_6_D_6_, 400 MHz): δ 8.06–7.92 (m, 2H), 7.73–7.58 (m, 2H), 7.14–7.08 (m, 3H), 7.07–7.03 (m, 2H), 4.36 (bd, 1H, ^2^ *J*_HF_ = 51.6 Hz), 2.47 (ddd, 1H, ^2^ *J*_HH_ = 15.7 Hz, ^3^ *J*_HH_ = 7.9 Hz, ^4^ *J*_HF_ = 3.4 Hz), 2.39 (ddd, 1H, ^2^ *J*_HH_ = 15.7 Hz, ^3^ *J*_HH_ = 7.8 Hz, ^4^ *J*_HF_ = 2.7 Hz), 1.04–0.91 (m, 1H), 0.29–0.15 (AA’BB’, 2H), 0.04-(−0.11) (AA’BB’, 2H). ^13^C­{^1^H} NMR (C_6_D_6_, 100 MHz): δ 160.7 (d, ^2^ *J*_CF_ = 20.6 Hz), 153.3 (^2^ *J*_CF_ = 19.8 Hz), 140.22, 140.17, 135.7 (d, ^3^ *J*_CF_ = 3.4 Hz), 130.7, 130.3, 130.2, 128.8, 128.7, 126.1, 125.8, 92.0 (bd, ^1^ *J*_CF_ = 202.3 Hz), 39.3, 8.7 (d, ^4^ *J*_CF_ = 2.1 Hz), 5.4, 5.0. ^13^C­{^1^H} NMR (CDCl_3_, 100 MHz): δ 161.6 (d, ^2^ *J*_CF_ = 21.1 Hz), 154.2 (d, ^2^ *J*_CF_ = 16.2 Hz), 139.7, 139.5, 135.1 (d, *J*_CF_ = 3.0 Hz), 130.8, 129.9 (d, *J*_CF_ = 6.3 Hz), 128.5, 128.4, 127.7, 126.1, 125.9, 91.7 (bd, ^1^ *J*_CF_ = 196.5 Hz), 39.4, 8.6 (d, ^4^ *J*_CF_ = 2.2 Hz), 5.3, 4.8. ^19^F­{^1^H} NMR (C_6_D_6_, 376 MHz): δ −198.3 (br s).

#### 4-(Cyclopropylmethyl)-3-fluoro-8,9-dimethyl-2-phenyl-3*H*-benzo­[*b*]­[1,4]­diazepine (**5cb**)

From **3c** (0.134 g, 0.663 mmol) and 4,5-dimethyl-1,2-phenylenediamine **4b** (0.0900g, 0.661 mmol), a yellow oil of **5cb** (0.145 g, 0.453 mmol, 68%). *R*_f_ = 0.55 (5:1 hexanes/ethyl acetate). HRMS (ESI-TOF) [M + H]^+^ calcd for C_21_H_21_FN_2_ 321.1762, found 321.1780. IR (cm^–1^, FTIR): 1635 s (C=N), 1616 m (C=N), 1295 s, 1105 s. ^1^H NMR (C_6_D_6_, 400 MHz): δ 8.07 (dt, 2H, ^3^ *J*_HH_ = 7.9 Hz, ^4^ *J*_HH_ = 1.5 Hz), 7.50 (s, 1H), 7.49 (s, 1H), 7.15–7.11 (m, 3H), 4.59 (bd, 1H, ^2^ *J*_HF_ = 51.1 Hz), 2.54 (ddd, 1H, ^2^ *J*_HH_ = 15.7 Hz, ^3^ *J*_HH_ = 7.9 Hz, ^4^ *J*_HF_ = 3.2 Hz), 2.44 (ddd, 1H, ^2^ *J*_HH_ = 15.7 Hz, ^3^ *J*_HH_ = 7.9 Hz, ^4^ *J*_HF_ = 2.5 Hz), 2.05 (s, 6H), 1.12–1.00 (m, 1H), 0.34–0.20 (AA’BB’, 2H), 0.09-(−0.04) (AA’BB’, 2H). ^13^C­{^1^H} NMR (C_6_D_6_, 100 MHz): δ 159.2 (d, ^2^ *J*_CF_ = 22.6 Hz), 152.0 (d, ^2^ *J*_CF_ = 18.6 Hz), 138.4­(2C), 135.9 (d, ^3^ *J*_CF_ = 3.2 Hz), 135.1, 134.8, 130.5, 130.2, 130.1, 129.2, 128.7, 92.2 (d, ^1^ *J*_CF_ = 210.4 Hz), 39.3, 19.4­(2C), 8.8 (d, ^4^ *J*_CF_ = 2.0 Hz), 5.4, 5.1. ^19^F­{^1^H} NMR (C_6_D_6_, 376 MHz): δ −198.0 (br s).

#### 4-(Cyclopropylmethyl)-3-fluoro-2-phenyl-3*H*-naphtho­[*b*]­[1,4]­diazepine (**5cd**)

From **3c** (0.1464 g, 0.7239 mmol) and *2,3*-diaminonaphthalene **4d** (0.1202 g, 0.7598 mmol), a yellow oil of **5 cd** (0.156 g, 0.456 mmol, 63%). *R*_f_ = 0.50 (hexanes/ethyl acetate 5:1). HRMS (ESI-TOF) [M + H]^+^ calcd for C_23_H_19_FN_2_ 343.1605, found 343.1605. IR (cm^–1^, FTIR): 1600 m (C=N), 1286 s, 1105 s. ^1^H NMR (C_6_D_6_, 400 MHz): δ 8.11 (s, 1H), 8.10 (s, 1H), 8.06–8.02 (m, 2H), 7.68–7.62 (m, 2H), 7.22–7.19 (m, 2H), 7.16–7.14 (m, 3H), 4.65 (d, 1H, ^2^ *J*_HF_ = 51.0 Hz), 2.54 (ddd, 1H, ^2^ *J*_HH_ = 15.1 Hz, ^3^ *J*_HH_ = 7.9 Hz, ^4^ *J*_HF_ = 3.3 Hz), 2.48 (ddd, 1H, ^2^ *J*_HH_ = 14.6 Hz, ^3^ *J*_HH_ = 7.8 Hz, ^4^ *J*_HH_ = 2.9 Hz), 1.12–1.00 (m, 1H), 0.36–0.22 (AA'BB', 2H), 0.10-(−0.02) (AA'BB', 2H). ^13^C­{^1^H} NMR (C_6_D_6_, 100 MHz): δ 165.8 (d, ^2^ *J*_CF_ = 23.2 Hz), 158.8 (d, ^2^ *J*_CF_ = 19.5 Hz), 139.5, 139.3, 136.0 (d, ^3^ *J*_CF_ = 3.0 Hz), 132.0, 131.8, 130.8, 130.0, 129.9, 128.7, 128.5, 126.6, 126.5, 126.3, 125.8, 90.9 (d, ^1^ *J*_CF_ = 201.3 Hz), 39.9, 8.6 (d, ^4^ *J*_CF_ = 2.2 Hz), 5.4, 5.1. ^19^F­{^1^H} NMR (C_6_D_6_, 376 MHz): δ −197.7 (br s).

#### 4-(4-*tert*-Butylbenzyl)-2-(4-chlorophenyl)-3-fluoro-8,9-dimethyl-3*H*-benzo­[*b*]­[1,4]­diazepine (**5db**)

From **3d** (0.0838 g, 0.255 mmol) and 4,5-dimethyl-1,2-phenylenediamine **4b** (0.0900g, 0.268 mmol), a yellow wax of **5db** (0.0739 g, 0.165 mmol, 65%). *R*_f_ = 0.55 (hexanes/ethyl acetate 6:1). HRMS (ESI-TOF) [M + H–CH3]^+^ calcd for C_27_H_27_ClFN_2_ 433.1841, found 433.1849. IR (cm^–1^, FTIR): 1704 m (C=N), 1592 m (C=N), 1295 s, 1107 s, 1014 s. ^1^H NMR (C_6_D_6_, 400 MHz): δ 7.53 (d, 2H, ^3^ *J*_HH_ = 8.0 Hz), 7.49 (s, 1H), 7.44 (s, 1H), 7.02 (s, 1H), 7.00 (s, 1H), (dt, 2H, ^3^ *J*_HH_ = 4.6 Hz, ^4^ *J*_HH_ = 2.0 Hz), 6.94 (d, 1H, ^4^ *J*_HH_ = 2.0 Hz), 4.54 (bd, 1H, ^2^ *J*_HF_ = 50.1 Hz), 3.96 (ddd, 1H, ^2^ *J*_HH_ = 20.1 Hz, ^4^ *J*_HF_ = 4.5 Hz), 3.90 (ddd, 1H, ^2^ *J*_HH_ = 19.9 Hz, ^4^ *J*_HF_ = 4.1 Hz), 2.04 (s, 6H), 1.14 (s, 9H). ^13^C­{^1^H} NMR (C_6_D_6_, 100 MHz): δ 158.3 (d, ^2^ *J*_CF_ = 22.0 Hz), 151.2 (d, ^2^ *J*_CF_ = 23.1 Hz), 149.7, 138.4, 138.1, 136.4, 135.5, 135.2, 133.6 (d, ^3^ *J*_CF_ = 2.9 Hz), 132.7, 131.4, 131.3, 129.4, 129.3, 128.9, 125.6, 91.5 (bd, ^1^ *J*_CF_ = 218.3 Hz), 40.9, 34.3, 31.4­(3C), 19.38, 19.35. ^19^F­{^1^H} NMR (C_6_D_6_, 376 MHz): δ −197.0 (br s).

#### (*E*)-3-[(3-Aminophenyl)­amino]-2-fluoro-4-(4-methylphenyl)-1-phenylbut-2-en-1-one (**8aa**)

A 50 mL round-bottom flask equipped with a stir bar was charged with fluoroalkynone **3a** (0.142 g, 0.563 mmol), *o*-phenylenediamine **4a** (0.610 g, 0.564 mmol), triethylamine (0.139 mL, 0.997 mmol), and ethanol (5 mL). The mixture was stirred at room temperature for 2 h. The solution was concentrated under reduced pressure. Silica gel flash column chromatography (15 × 2.5 cm; hexanes/ethyl acetate 80:20 v/v) gave a dark oil of **8aa** (0.161 g, 0.447 mmol, 80%). HRMS (ESI-TOF) [M + H]^+^ calcd for C_23_H_21_FN_2_O 361.1711, found 361.1709. IR (cm^–1^, FTIR): 3073 w (N–H), 3031 w (N–H), 1608 s (C=O), 1578 (C=C), 1542 m (C=C), 1304 s, 1104 s. Tautomers - *italic* = *enamine*, underlined = imine. *) This peak includes the two hydrogens of the enamine and imino forms. ^1^H NMR (C_6_D_6_, 400 MHz): δ *11.73 (s, 1H), 8.25 (AA’XX’, 2H, J = 7.7 Hz)*, 8.20 (AA’XX’, 2H,*J*= 7.0, 1.4), *7.84 (d, 1H, J = 7.9 Hz), 6.92 (d, 3H, J = 7.7 Hz), 6.86 (d, 4H, J = 7.0, Hz)*, 6.70 (d, 4H,*J*= 7.8 Hz), *6.63 (d, 1H, *J* = 7.0, Hz), *6.42 (dt, 1H, *J* = 7.6, 1.2 Hz), *6.21 (dd, 1H, *J* = 8, 1.1 Hz), 4.26 (d, 2H, ^2^ *J* _HF_ = 47.7 Hz), 3.84 (d, 2H,*J*= 8.8 Hz, ^4^ *J*= 4.3 Hz), *3.70 (d, 2H,* ^4^ *J = 4.3 Hz)*, *3.00 (br s, 2H), *2.05 (s, 3H)*, 1.98 (s, 3H). ^13^C­{^1^H} NMR (C_6_D_6_, 100 MHz): δ 184.5, 184.3, 154.0 (d,*J*= 26.3 Hz), 143.9, 143.6, 141.4, 140.4, 139.9, 139.7, 138.5 (d, ^3^ *J*_CF_ = 6.2 Hz), 136.22, 136.18, 135.2, 134.0, 133.0, 132.7, 131.5, 131.3, 130.4, 130.2, 130.1, 129.75, 129.68, 129.5, 129.4, 129.3, 128.95, 128.87 (d, ^4^ *J* = 1.5 Hz), 128.7, *126.1 (d, J = 7.1 Hz), 123.7, 118.1, 115.8, 93.0 (d, J = 188.4 Hz)*, 41.0, 32.1, 32.03, 31.96, 30.2, 23.0, 21.2, 21.00, 20.96. ^19^F­{^1^H} NMR (C_6_D_6_, 376 MHz): δ −169.0 (s).

#### Synthesis of 3-Fluoro-4-(4-methylbenzyl)-2-phenyl-3*H*-benzo­[*b*]­[1,4]­diazepine (**5aa**) from Fluorinated Enaminone **8aa**

A 50 mL round-bottom flask equipped with a stir bar was charged with crude fluorinated enaminone **8aa** (0.200 g, 0.555 mmol) and a solution of acetic acid in ethanol (2% v/v; 20 mL). The reaction mixture was stirred at rt for 3 h. The solvent was removed by rotary evaporator followed by an oil pump vacuum to give **5aa**. Silica gel flash column chromatography (19 × 2.5 cm; hexanes/ethyl acetate 80:20) afforded **5aa** as a yellow oil (0.158 g, 0.462 mmol, 83%). Spectral data were identical to those described above.

### Computational

Initially, all structures involved in Figure 5 were built using Spartan’24.^39^ Next, the most stable conformation of each structure was determined using the Global Optimizer Algorithm (GOAT)^40^ with the GFN2-xTB Hamiltonian^41^ as implemented in the ORCA program suite^42^ and integrated with the GFN2-xTB libraries.^43^ This approach enabled a rapid search for the optimal conformation without resorting to molecular dynamics simulations. Finally, geometry optimization and vibrational analysis were performed for the identified conformers at the DFT level. A combination of universally reliable general-purpose exchange-correlation functional ωB97X-D^100^ developed by the Head-Gordon group and the DFT-specific polarization-consistent aug-pcseg-1 basis set developed by Jensen^101^ was employed. This choice of the basis set allowed for a favorable balance between minimizing the basis set incompleteness error and avoiding numerical linear dependencies or unreasonable computation time. Since the actual synthesis was performed in ethanol, the SMD implicit-solvent model was employed to account for the effects of the solvent on the properties of all species.^44^ All DFT simulations were performed using the Gaussian 16 program.^45^

## Supplementary Material





## Data Availability

The data underlying this study are available in the published article and its Supporting Information.

## Notes

^1^H and ^13^C­{^1^H} NMR spectra for fluoroalkynone **3b**, diazepines **5,** and enaminone **8aa** (PDF)
Computational data: Gaussian output files and a table of computed Gibbs energies for all reaction intermediates and transition states (ZIP)

## ⊥

Department of Chemistry, Natural Sciences Division, University of La Verne, 1950 Third Street, La Verne, California 91750, United States

## #

Materials Synthesis Group, Poznań Science and Technology Park, Rubież 46, 61–612 Poznań, Poland
