# Supplementary material for: Non-Catalyzed Cascade Double Imination Reaction of 2‑Fluoro-alk-3-yn-1-ones: Sustainable Synthesis of 3‑Fluoro-1,5-benzodiazepines
Source: ACS Org Inorg Au. 2026 Jan 30;6(2):237–47. doi: 10.1021/acsorginorgau.5c00116 (PMC13047452; doi:10.1021/acsorginorgau.5c00116)
Supplement: Supplementary file 1 [file gg5c00116_si_001.pdf]

SUPPORTING INFORMATION

Non-Catalyzed Cascade Double Imination Reaction of 2-Fluoro-alk-3-yn-1-ones: Sustainable Synthesis of 3-Fluoro-1,5-benzodiazepines

Trevor L. Olson,[a] Ariela W. Kaspi-Kaneti,[a],#$ Adrian Zając,[a],§$ Dominic Agyei Gyimah,[a] Austin Walsh,[a] Jacob A. Weston,[a] Alexander A. Rusakov,[a] Kraig A. Wheeler,[c] Béla Török,[d] and Roman Dembinski*,[a,b]

[a] *Department of Chemistry, Oakland University, 146 Library Drive, Rochester, Michigan 48309-4479, USA*
[b] *Centre of Molecular and Macromolecular Studies, Polish Academy of Sciences, Sienkiewicza 112, 90-363 Łódź, Poland*
[c] *Department of Chemistry, Whitworth University, 300 W. Hawthorne Rd., Spokane, WA 99251, USA.*
[d] *Department of Chemistry, University of Massachusetts Boston, 100 Morrissey Blvd., Boston, Massachusetts 02125, USA.*

Email Address: dembinsk@oakland.edu

Table of Contents

^{1}H and ^{13}C{^{1}H} NMR spectrum of **3b** S2–S3
^{1}H and ^{13}C{^{1}H} NMR spectrum of **5aa** S4–S5
^{1}H and ^{13}C{^{1}H} NMR spectrum of **5ab** S6–S7
^{1}H and ^{13}C{^{1}H} NMR spectrum of **5ac** S8–S9
^{1}H and ^{13}C{^{1}H} NMR spectrum of **5ad** S10–S11
^{1}H and ^{13}C{^{1}H} NMR spectrum of **5ba** S12–S13
^{1}H and ^{13}C{^{1}H} NMR spectrum of **5bb** S14–S16
^{1}H and ^{13}C{^{1}H} NMR spectrum of **5ca** S17–S18
^{1}H and ^{13}C{^{1}H} NMR spectrum of **5cb** S19–S20
^{1}H and ^{13}C{^{1}H} NMR spectrum of **5cd** S21–S22
Expansions of ^{1}H NMR cyclopropyl signals of **5ca**, **5cb**, and **5cd** S23
^{1}H and ^{13}C{^{1}H} NMR spectrum of **5db** S24–S25
^{1}H and ^{13}C{^{1}H} NMR spectrum of **8aa** S26–S27
Crystallography for **5aa** S28–S29

ACS Organic & Inorganic Au **2026**, *6*, doi.org/10.1021/acsorginorgau.5c00116

^{1}H NMR spectrum for **3b** ($CDCl_3$)

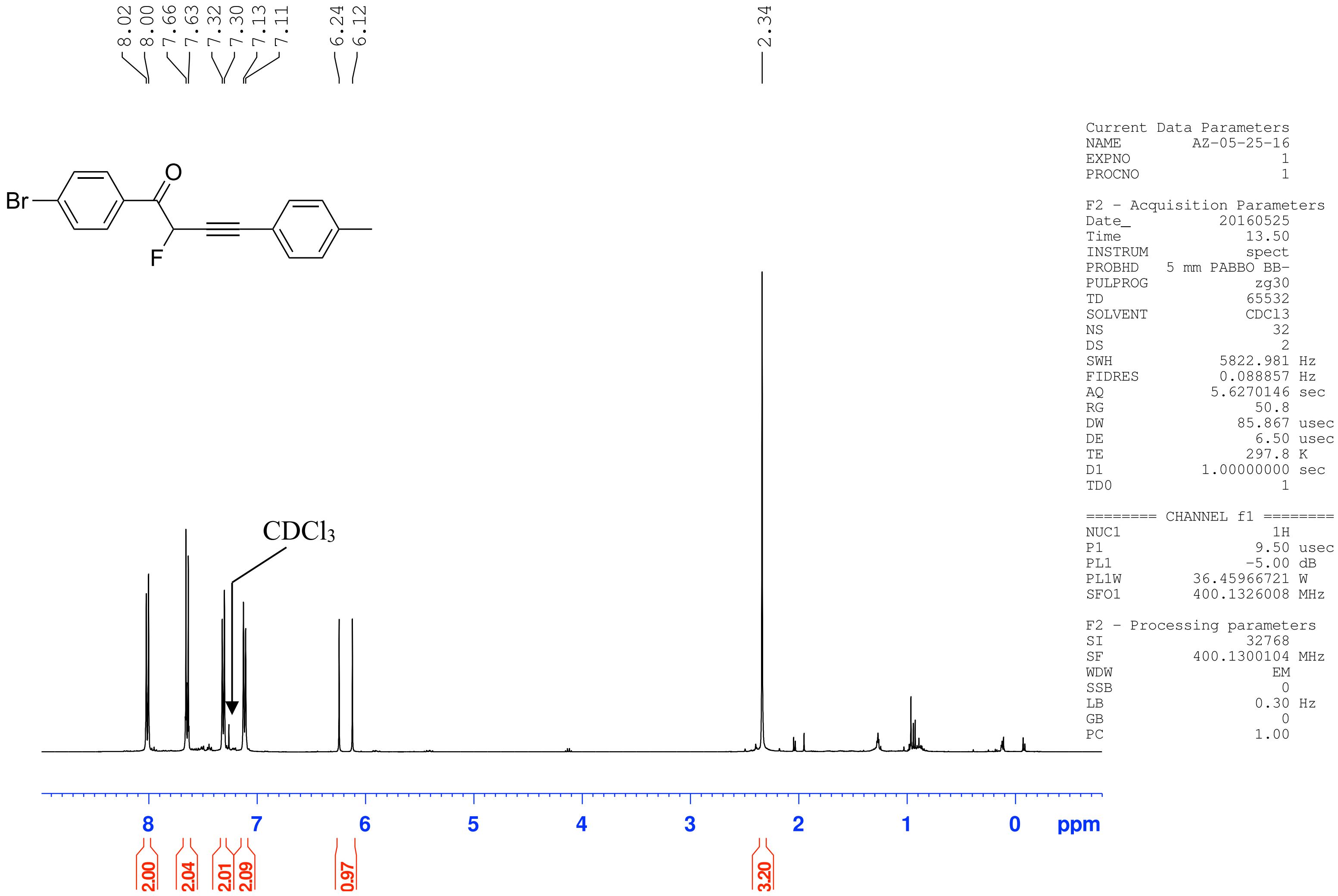

$^{13}C\{^{1}H\}$ NMR spectrum for **3b** ($CDCl_3$)

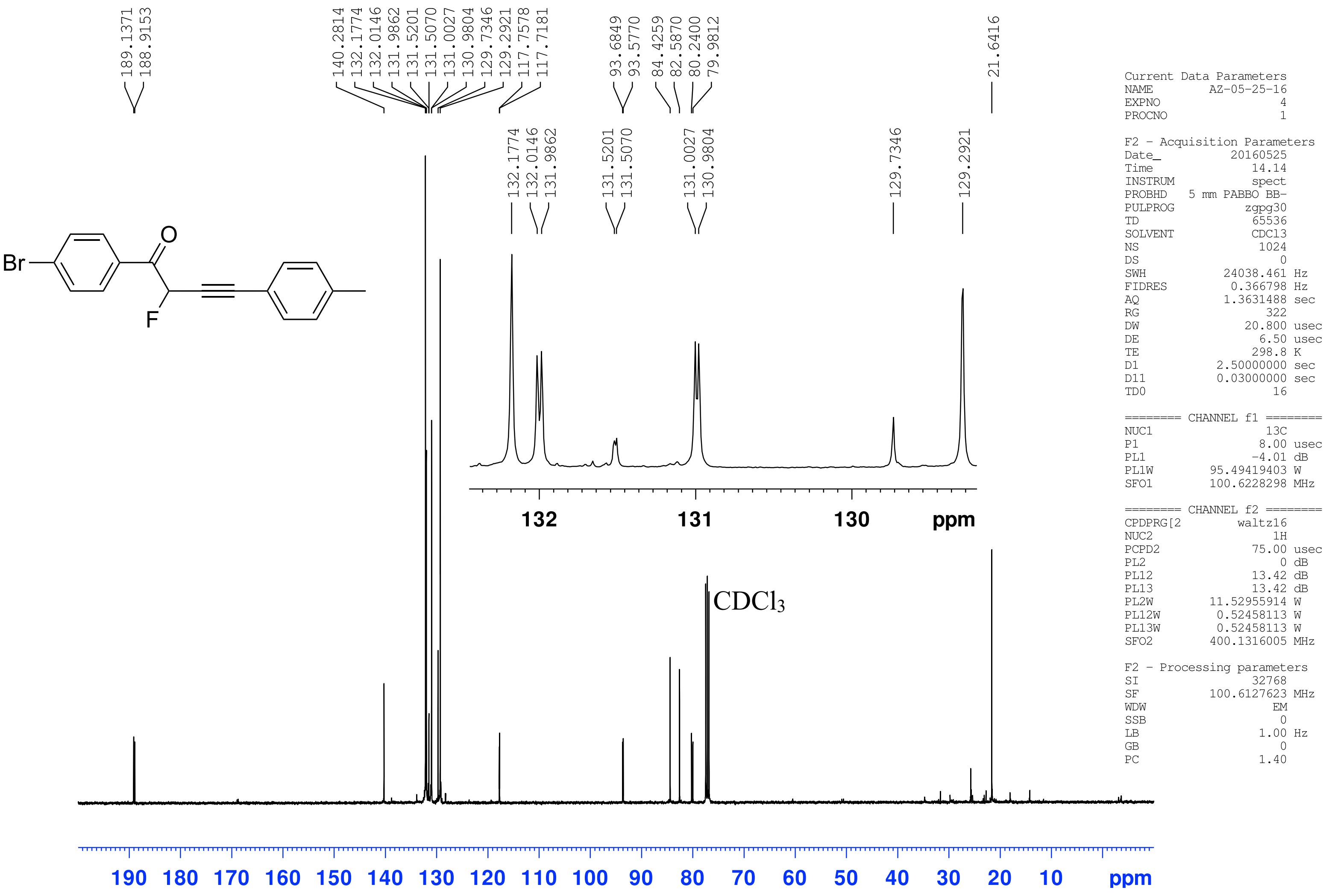

^{1}H NMR spectrum for **5aa** (C_6D_6)

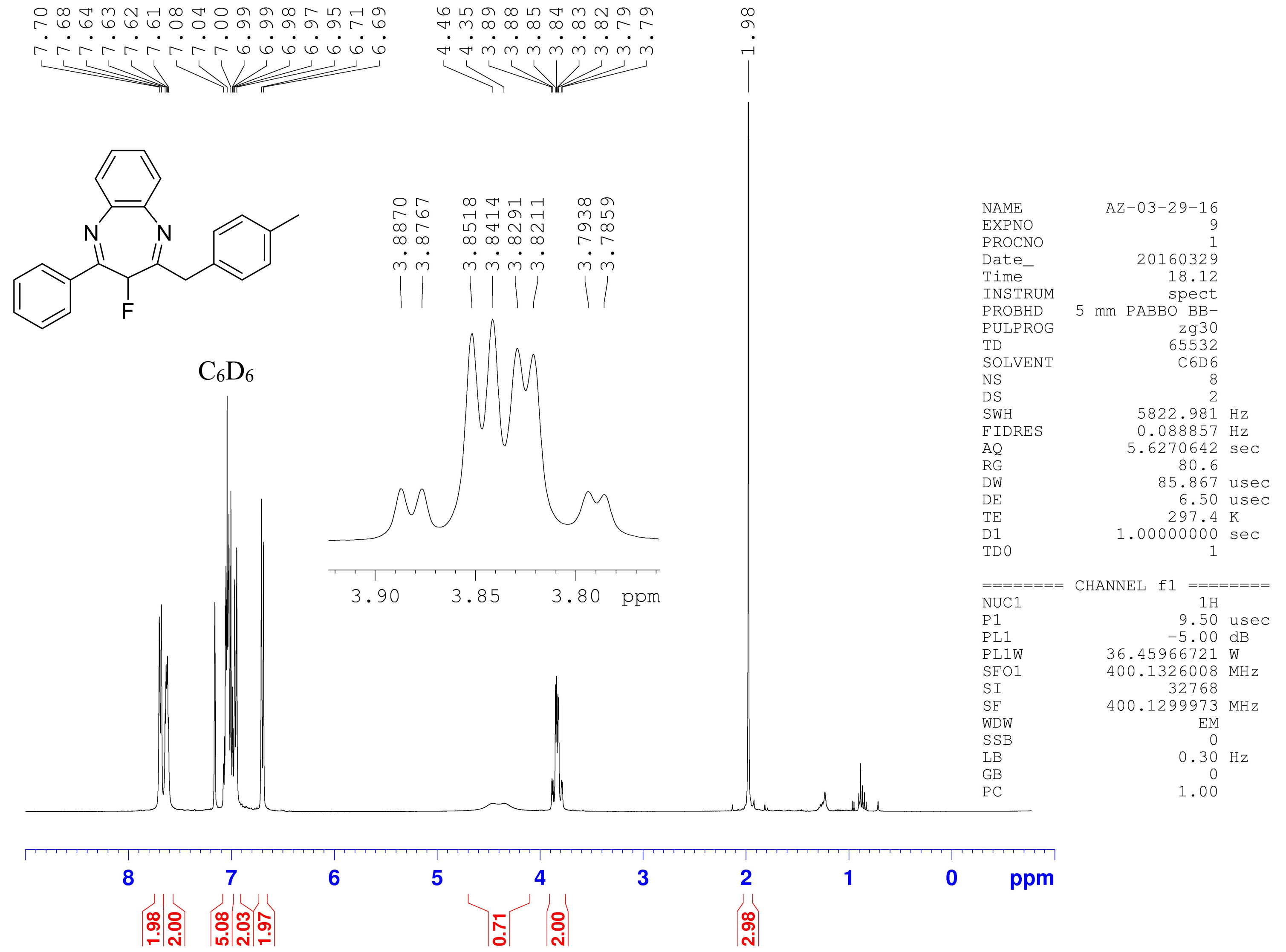

$^{13}C\{^{1}H\}$ NMR spectrum for **5aa** (C_6D_6)

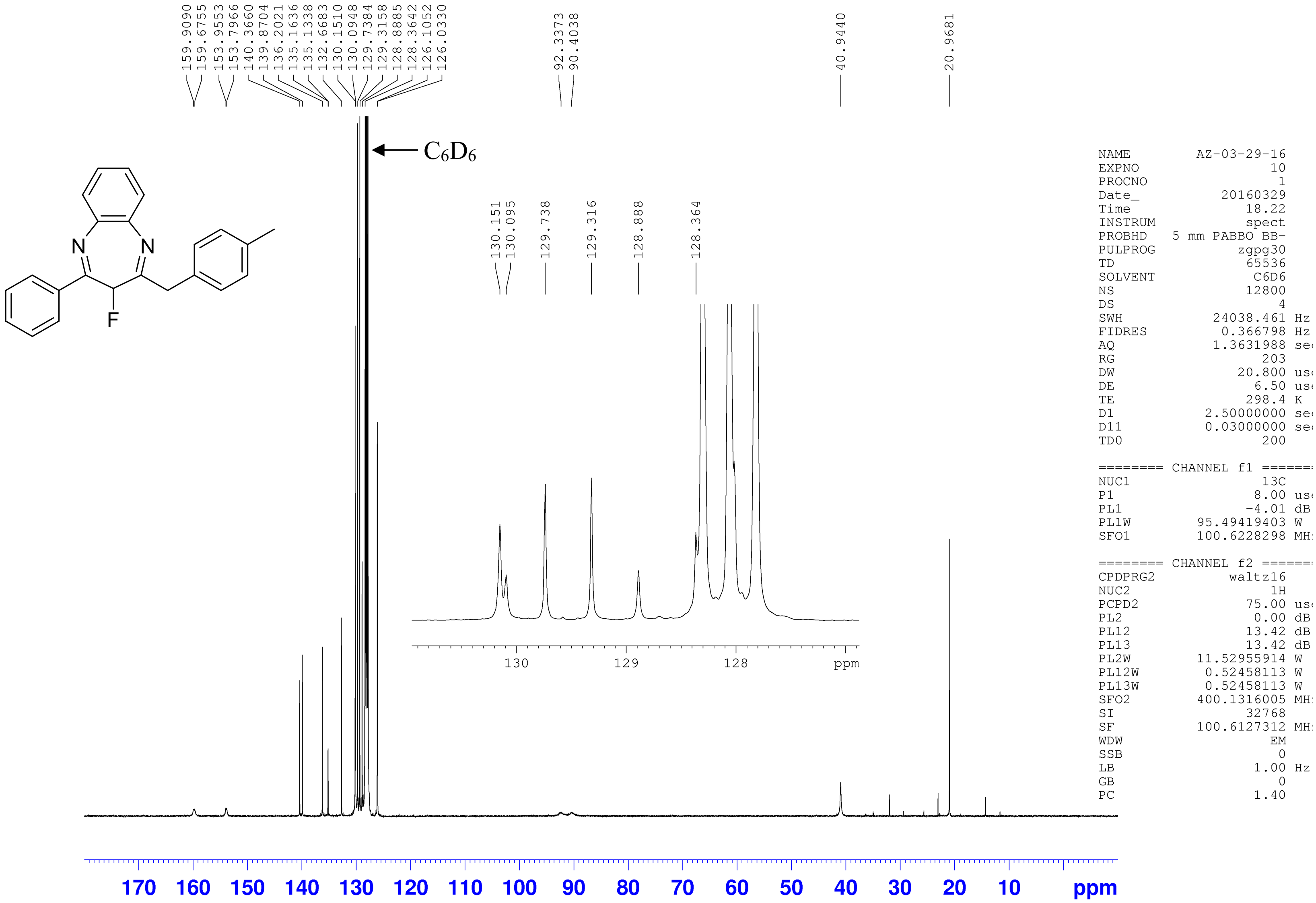

^{1}H NMR spectrum for **5ab** (C_6D_6)

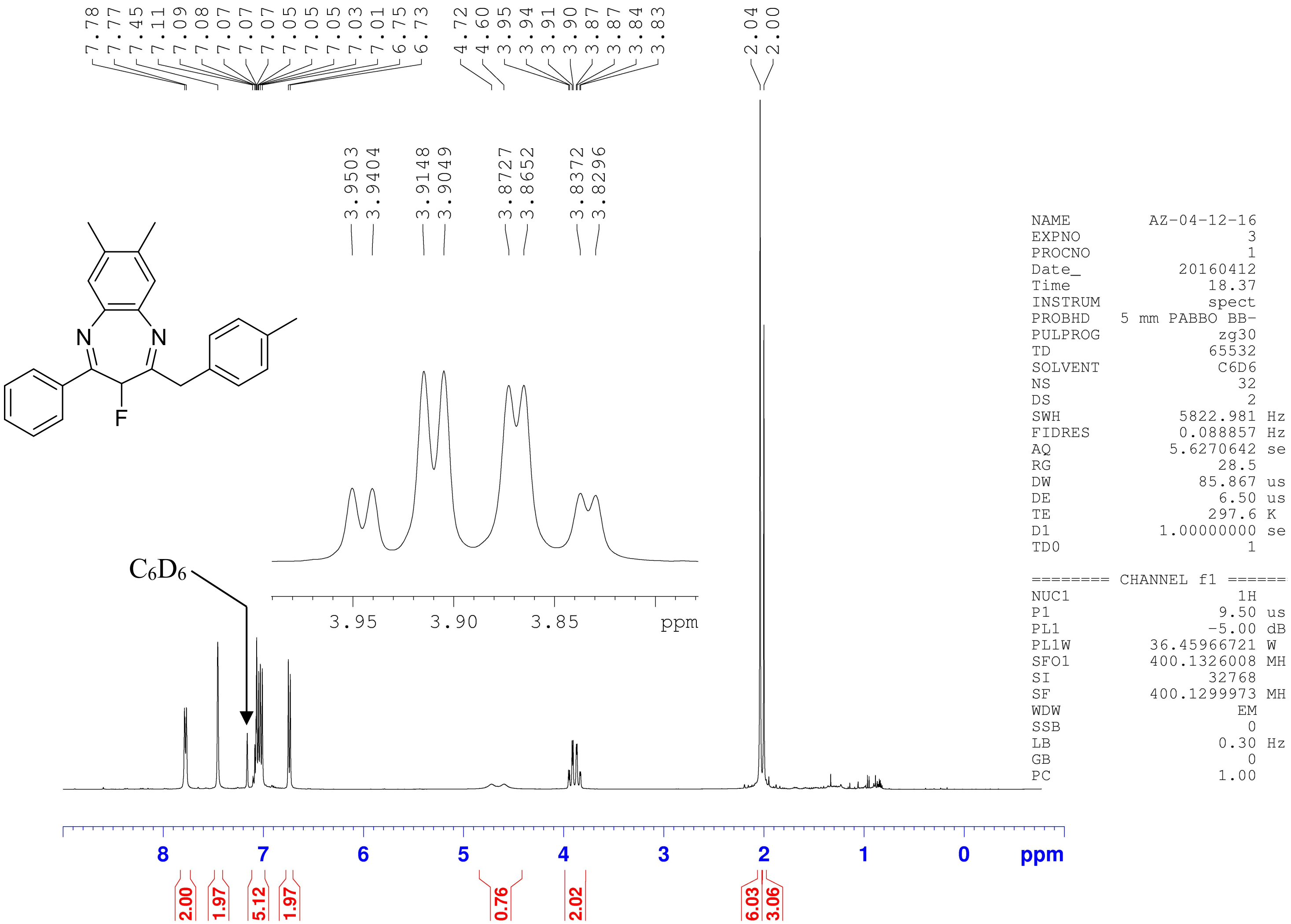

$^{13}C\{^{1}H\}$ NMR spectrum for **5ab** (C_6D_6)

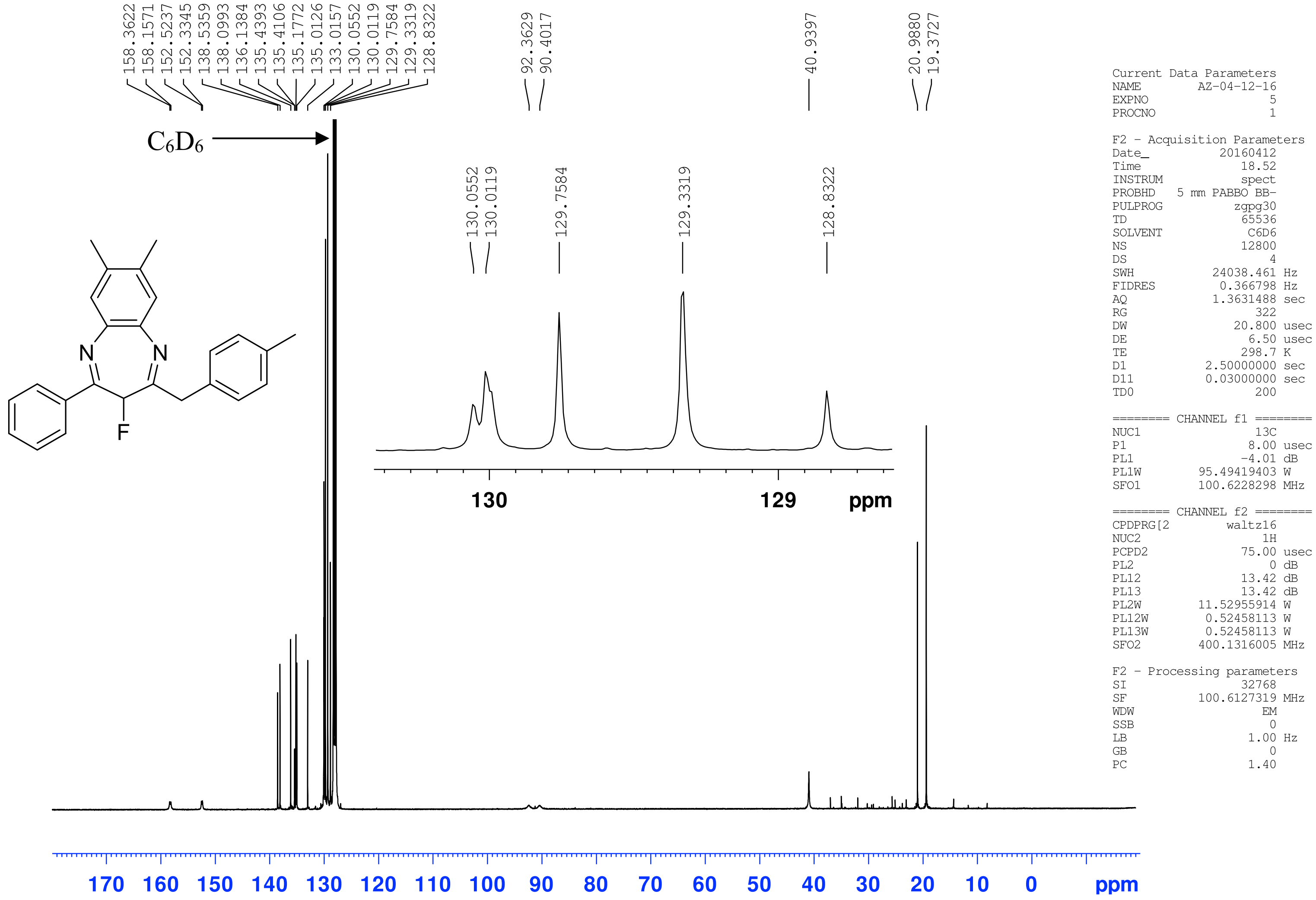

^{1}H NMR spectrum for **5ac** (C_6D_6)

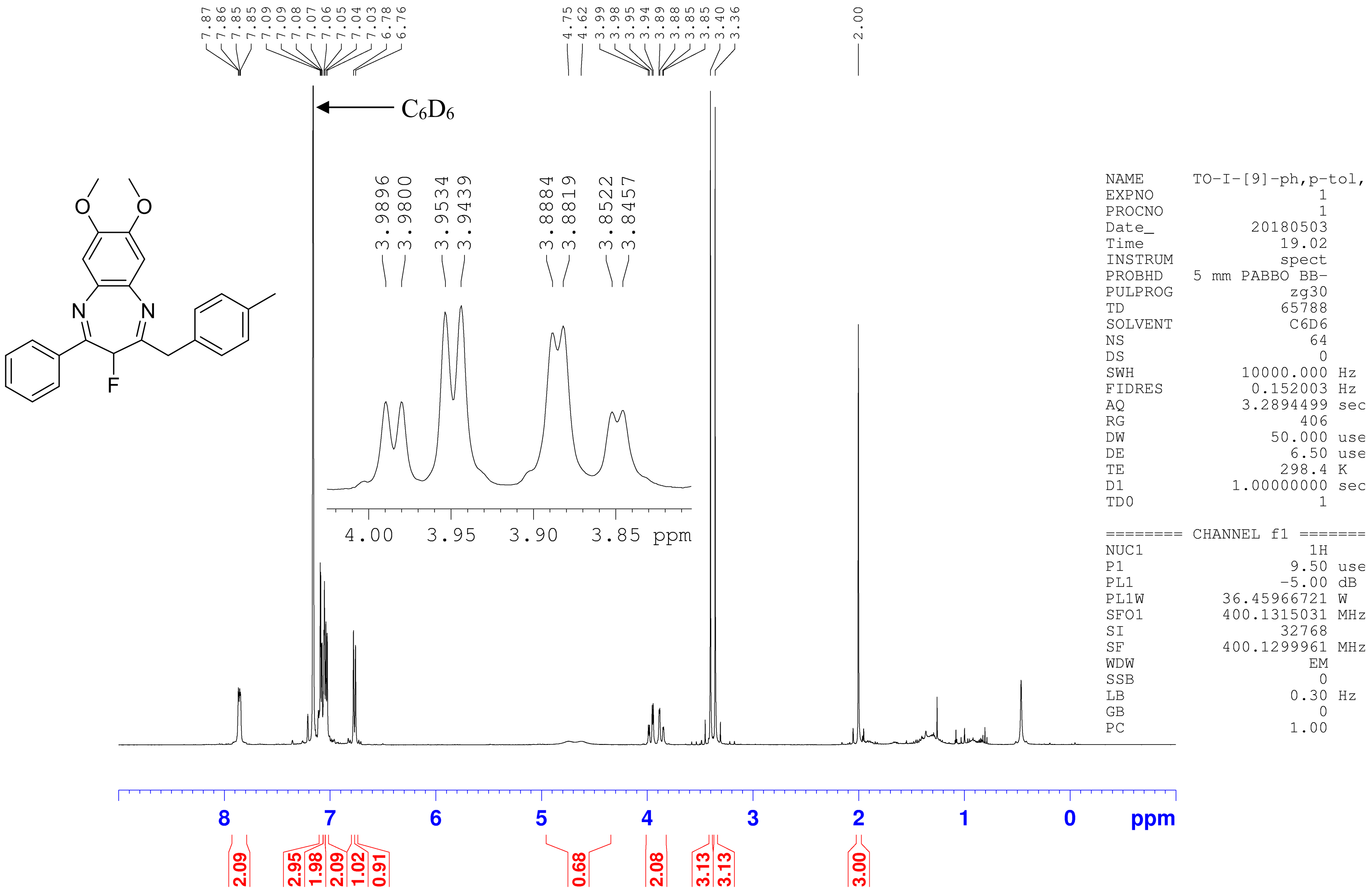

$^{13}C\{^{1}H\}$ NMR spectrum for **5ac** (C_6D_6)

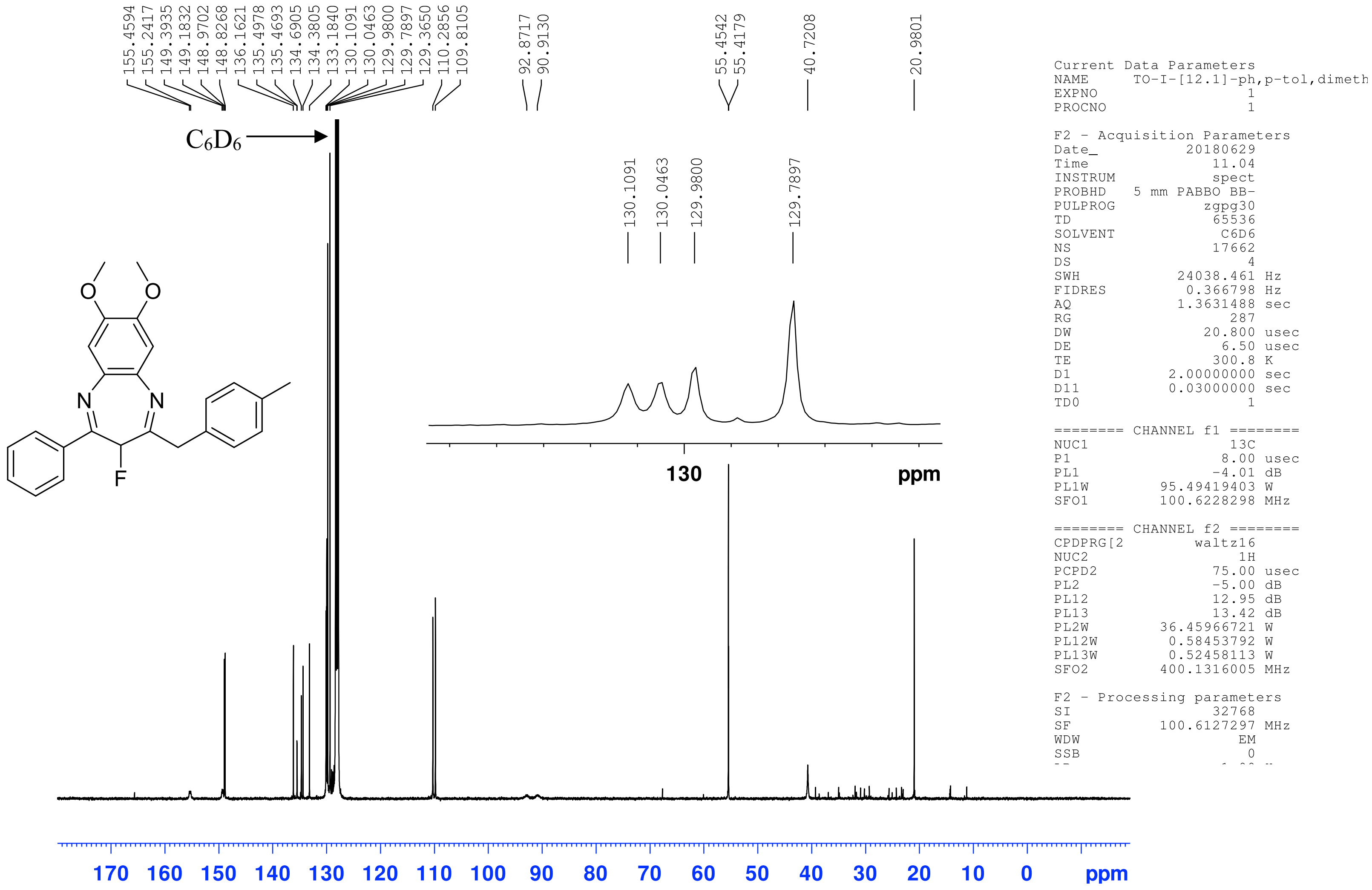

ACS Org. Inorg. Au **2026**, *6*, doi.org/10.1021/acsorginorgau.5c00116

^{1}H NMR spectrum for **5ad** (C_6D_6)

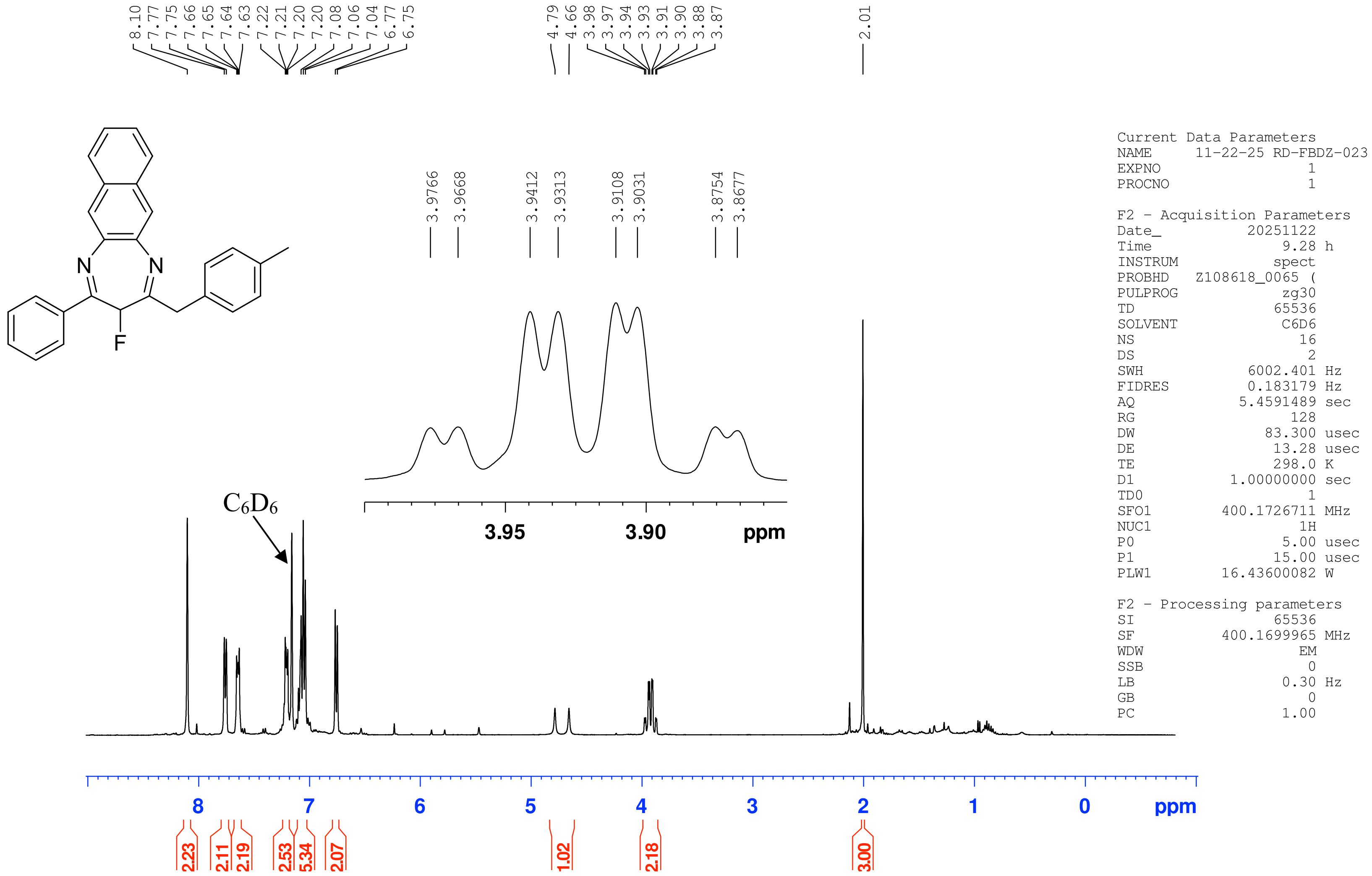

ACS Org. Inorg. Au **2026**, *6*, doi.org/10.1021/acsorginorgau.5c00116

$^{13}C\{^1H\}$ NMR spectrum for **5ad** (C_6D_6)

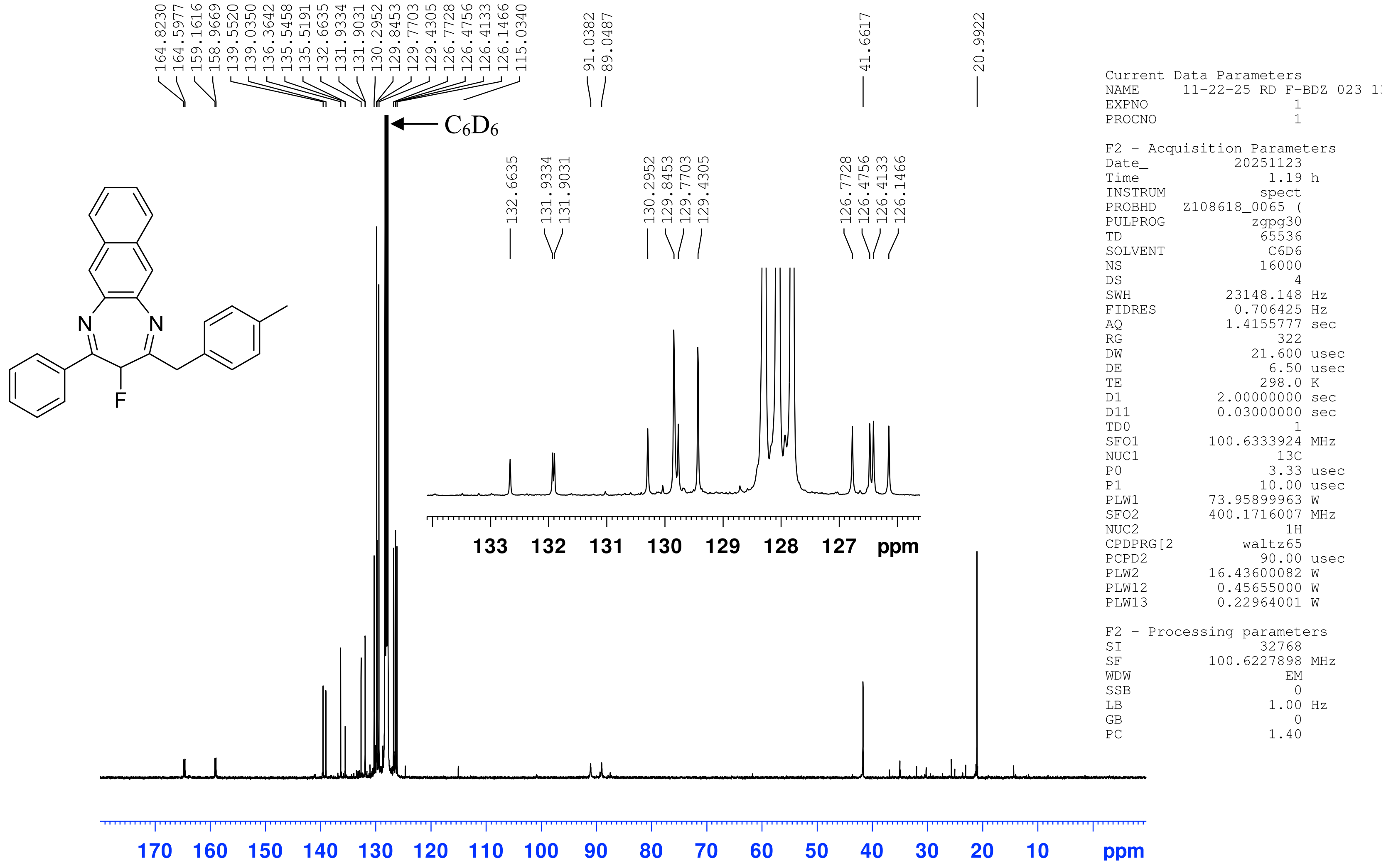

^{1}H NMR spectrum for **5ba** (C_6D_6)

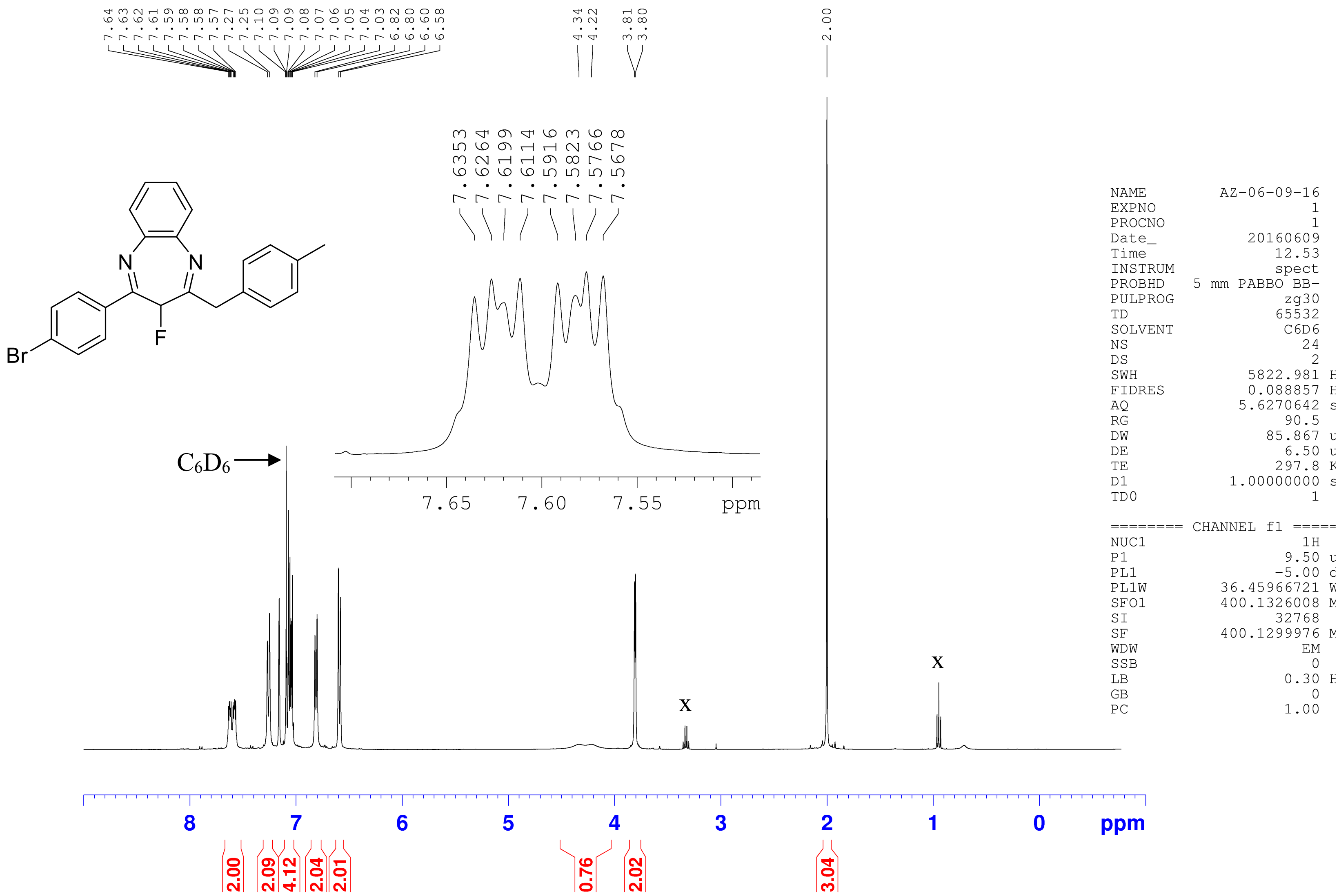

ACS Org. Inorg. Au **2026**, *6*, doi.org/10.1021/acsorginorgau.5c00116

$^{13}C\{^{1}H\}$ NMR spectrum for **5ba** (C_6D_6)

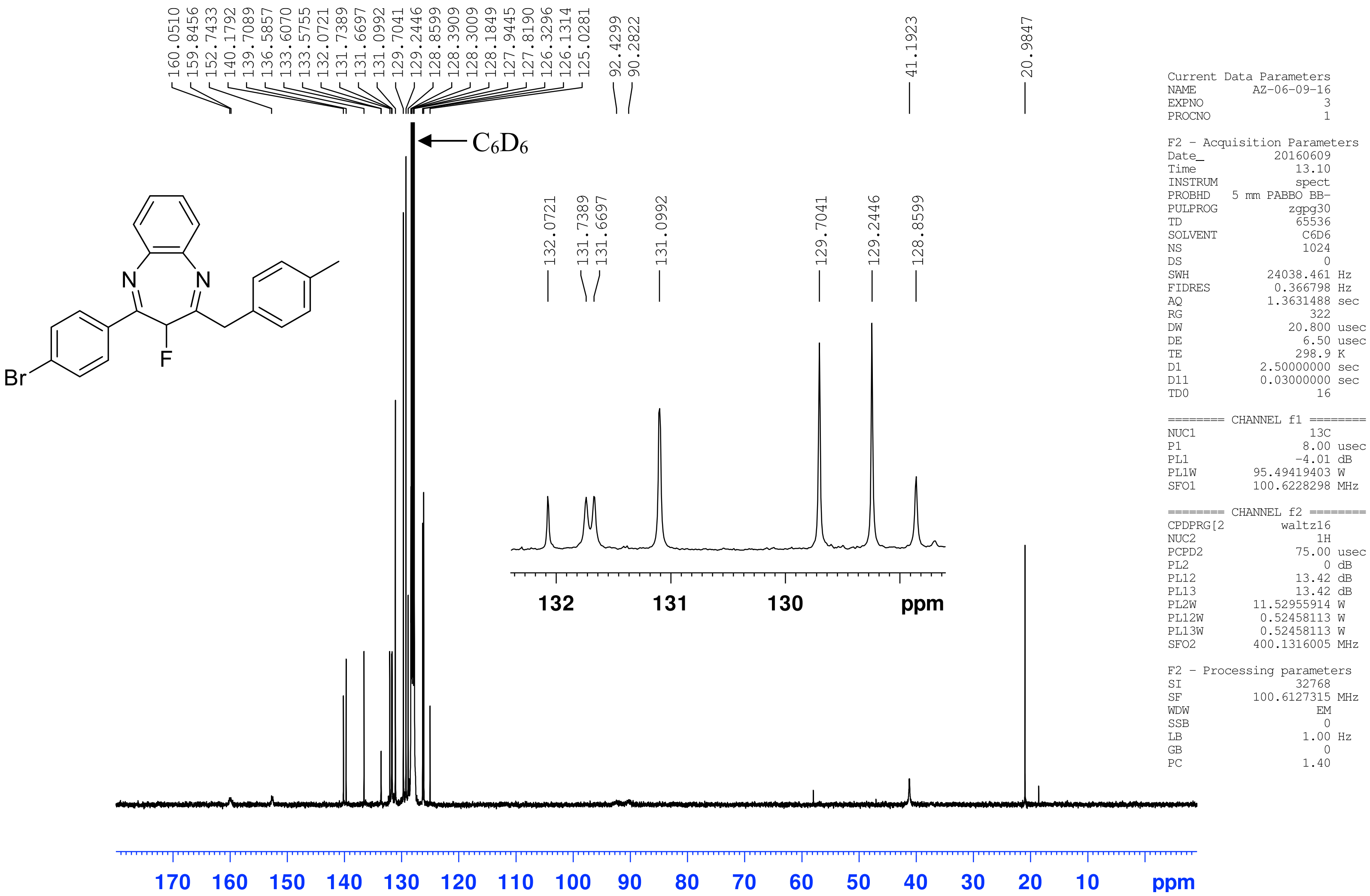

^{1}H NMR spectrum for **5bb** (C_6D_6)

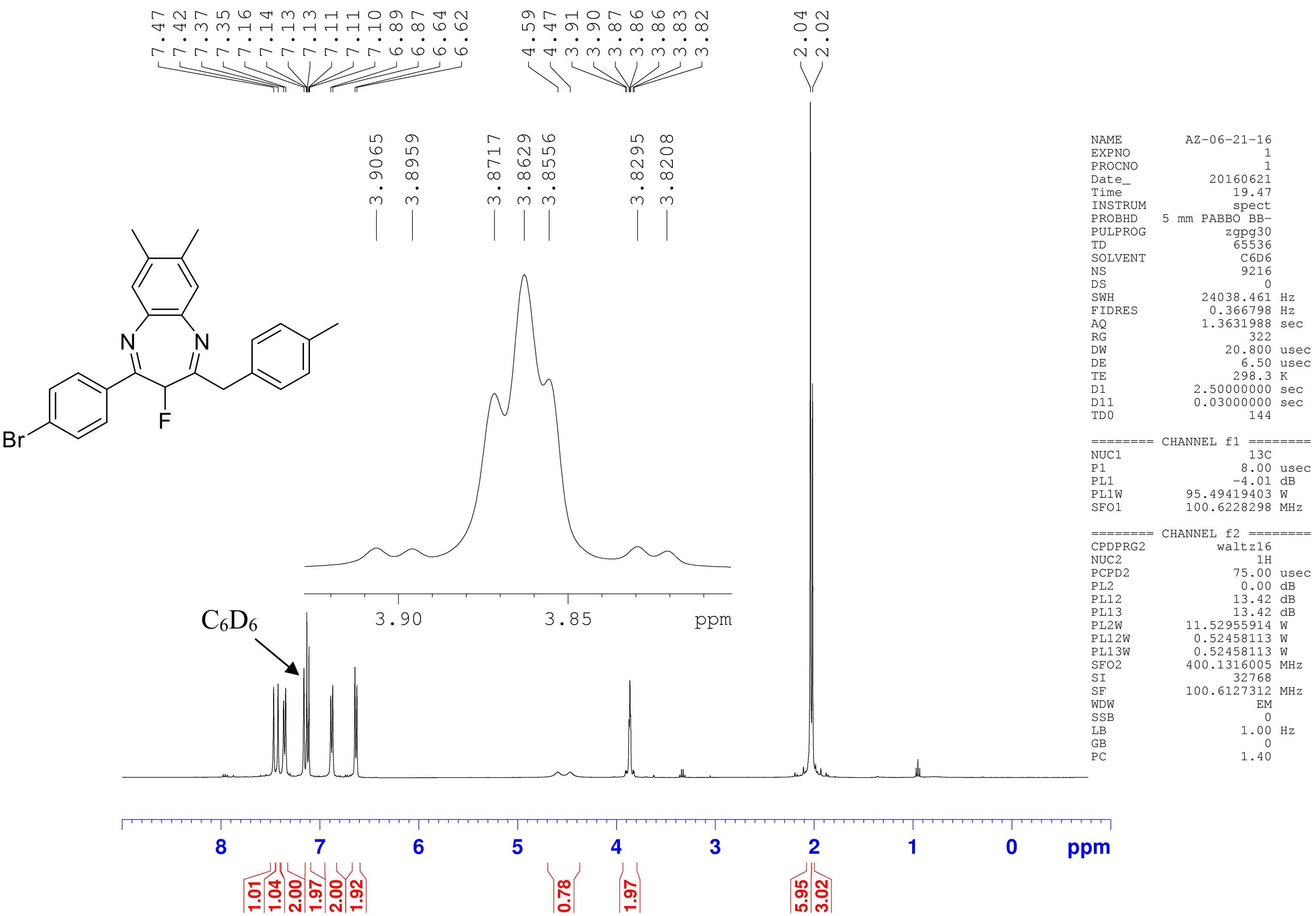

ACS Org. Inorg. Au **2026**, *6*, doi.org/10.1021/acsorginorgau.5c00116

$^{13}C\{^{1}H\}$ NMR spectrum for **5bb** (C_6D_6)

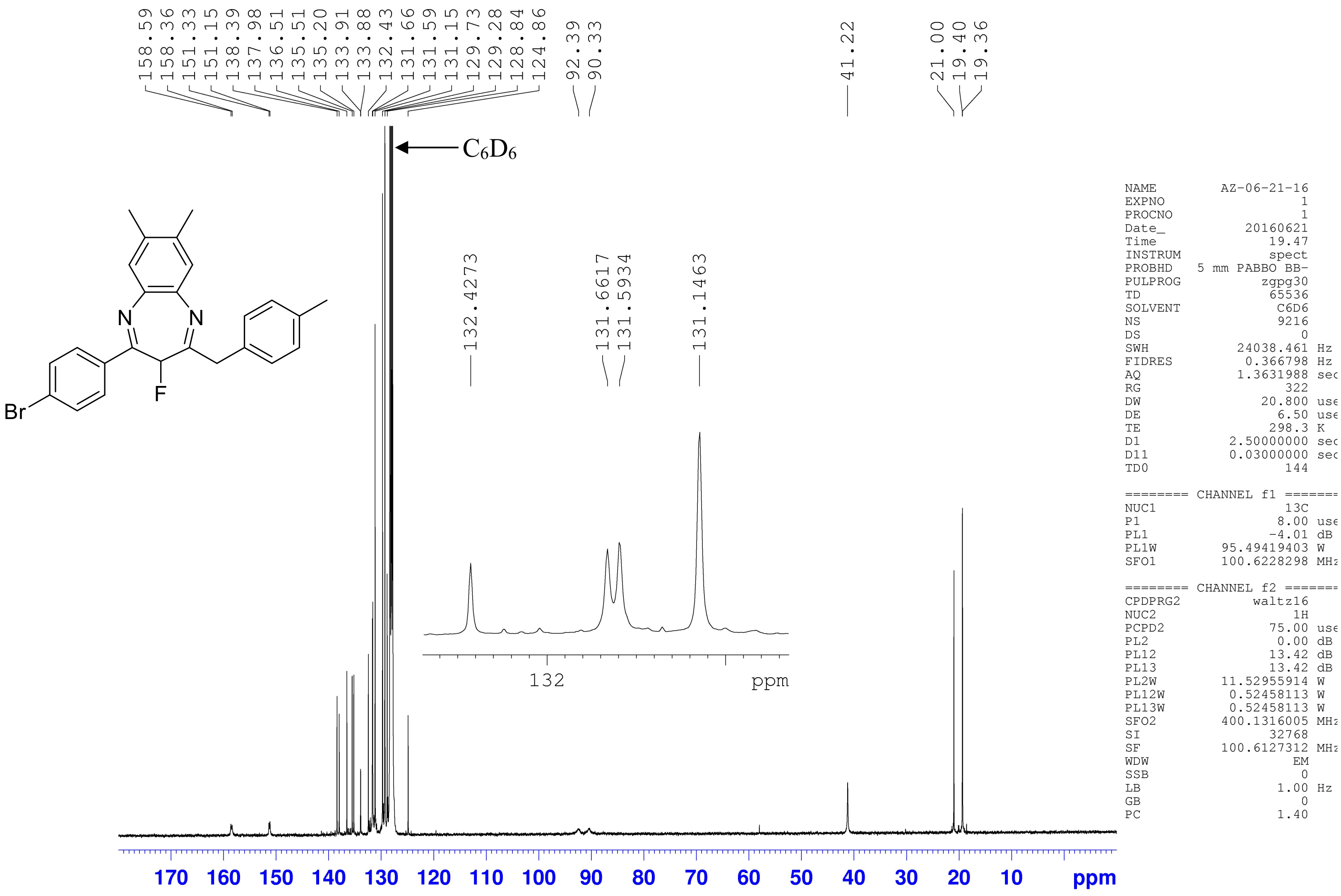

^{1}H NMR spectrum for **5ca** (C_6D_6)

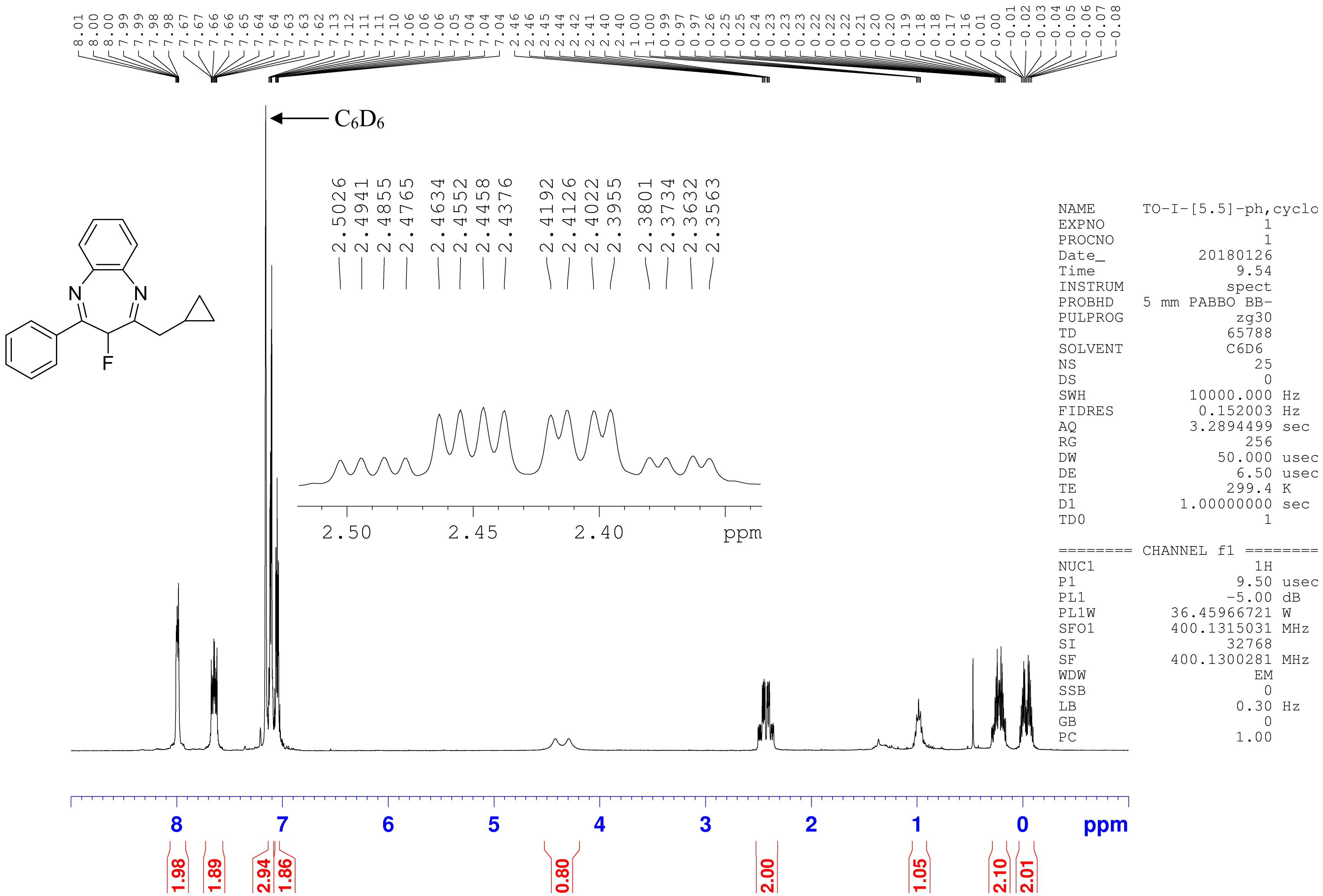

$^{13}C\{^{1}H\}$ NMR spectrum for **5ca** (C_6D_6)

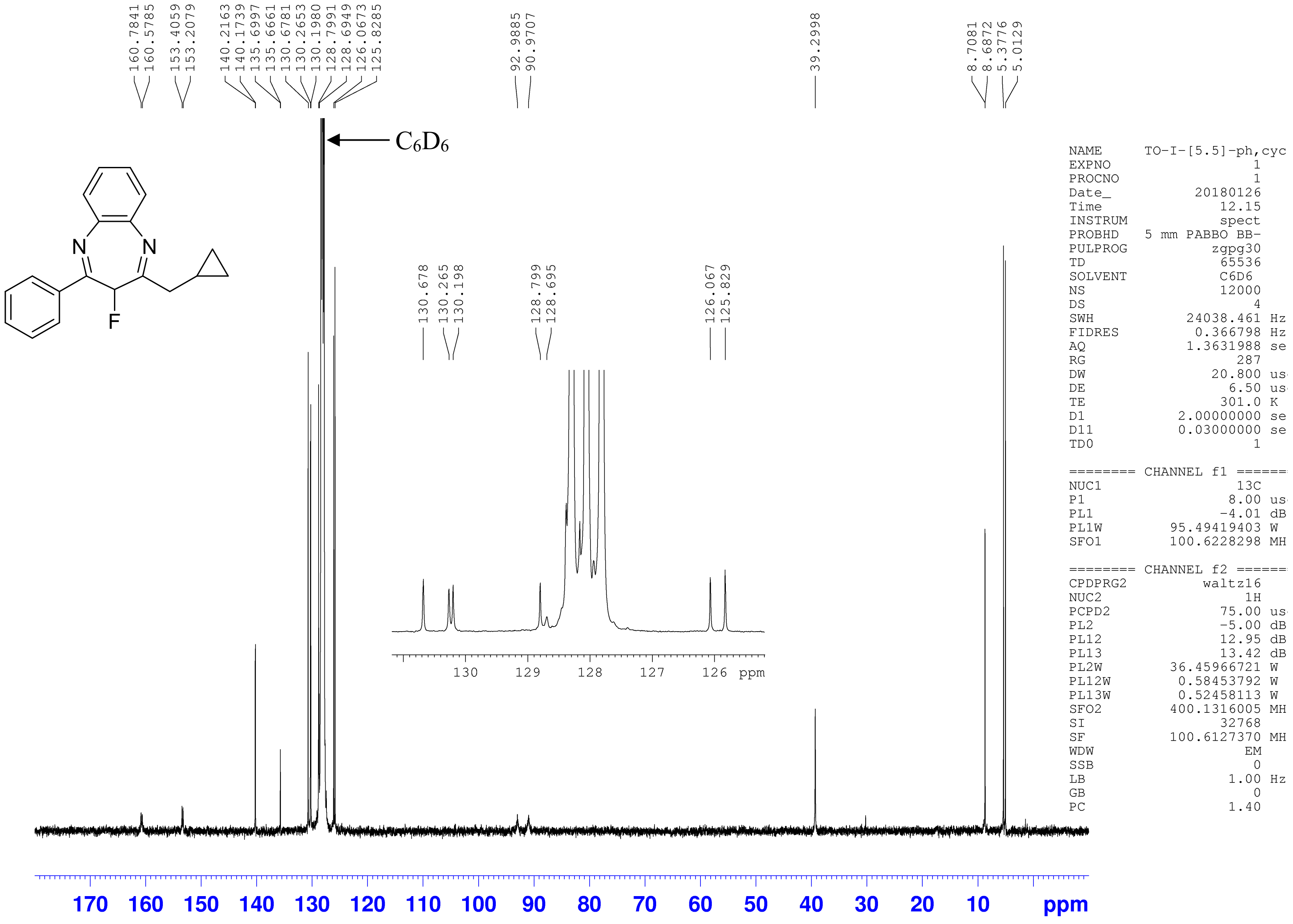

ACS Org. Inorg. Au **2026**, *6*, doi.org/10.1021/acsorginorgau.5c00116

$^{13}C\{^{1}H\}$ NMR spectrum for **5ca** ($CDCl_3$)

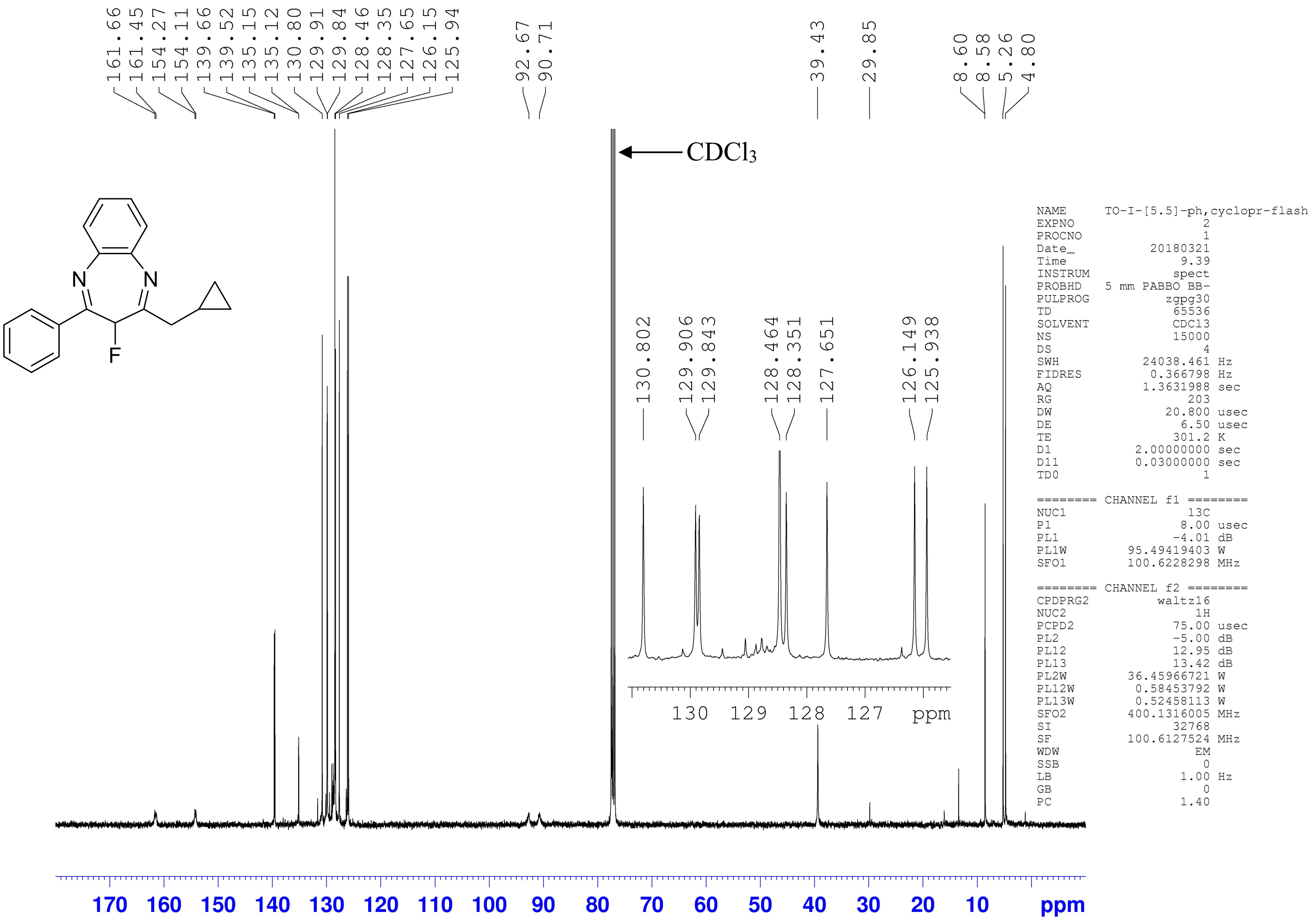

ACS Org. Inorg. Au **2026**, *6*, doi.org/10.1021/acsorginorgau.5c00116

^{1}H NMR spectrum for **5cb** (C_6D_6)

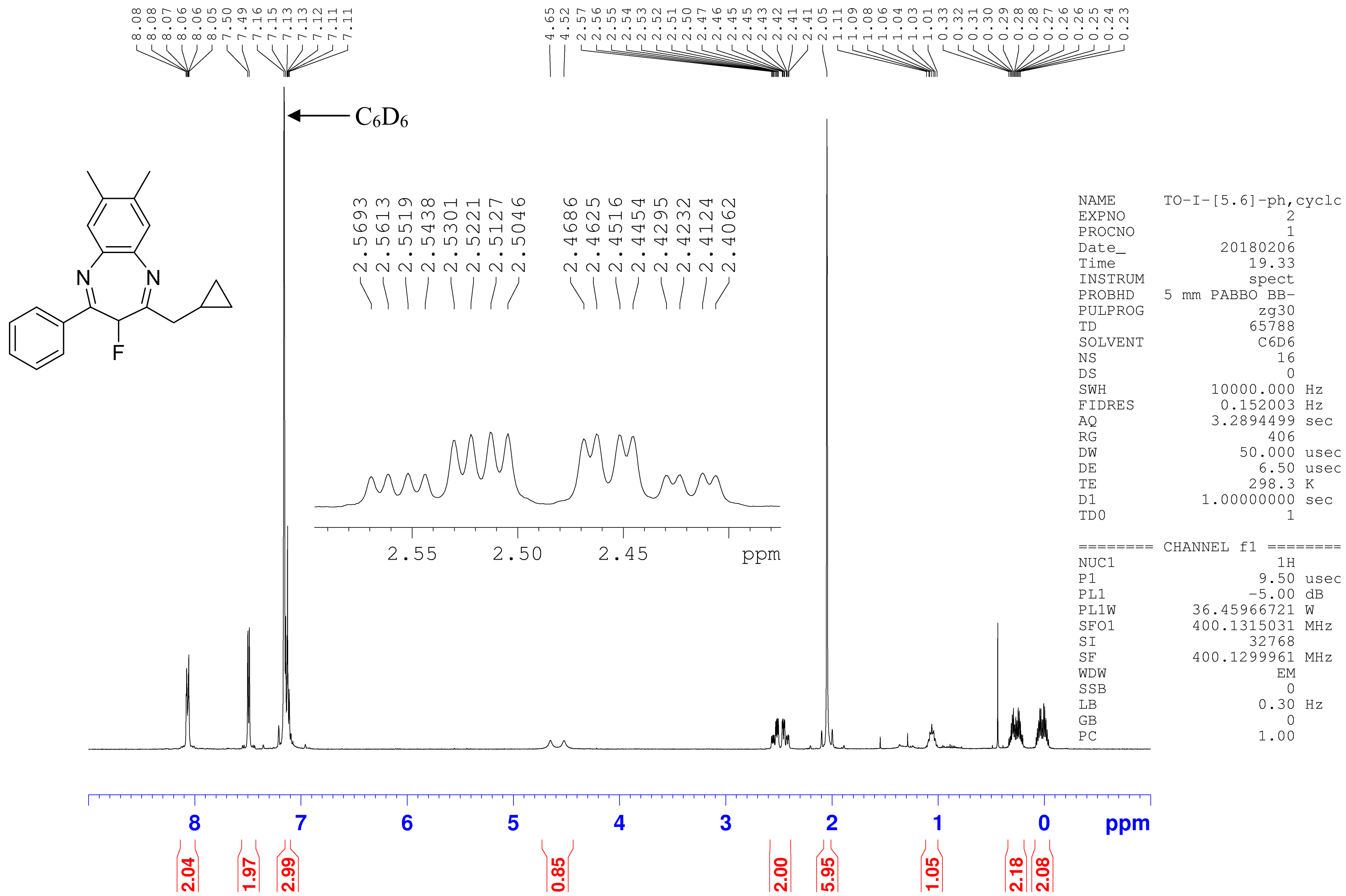

ACS Org. Inorg. Au **2026**, *6*, doi.org/10.1021/acsorginorgau.5c00116

$^{13}C\{^{1}H\}$ NMR spectrum for **5cb** (C_6D_6)

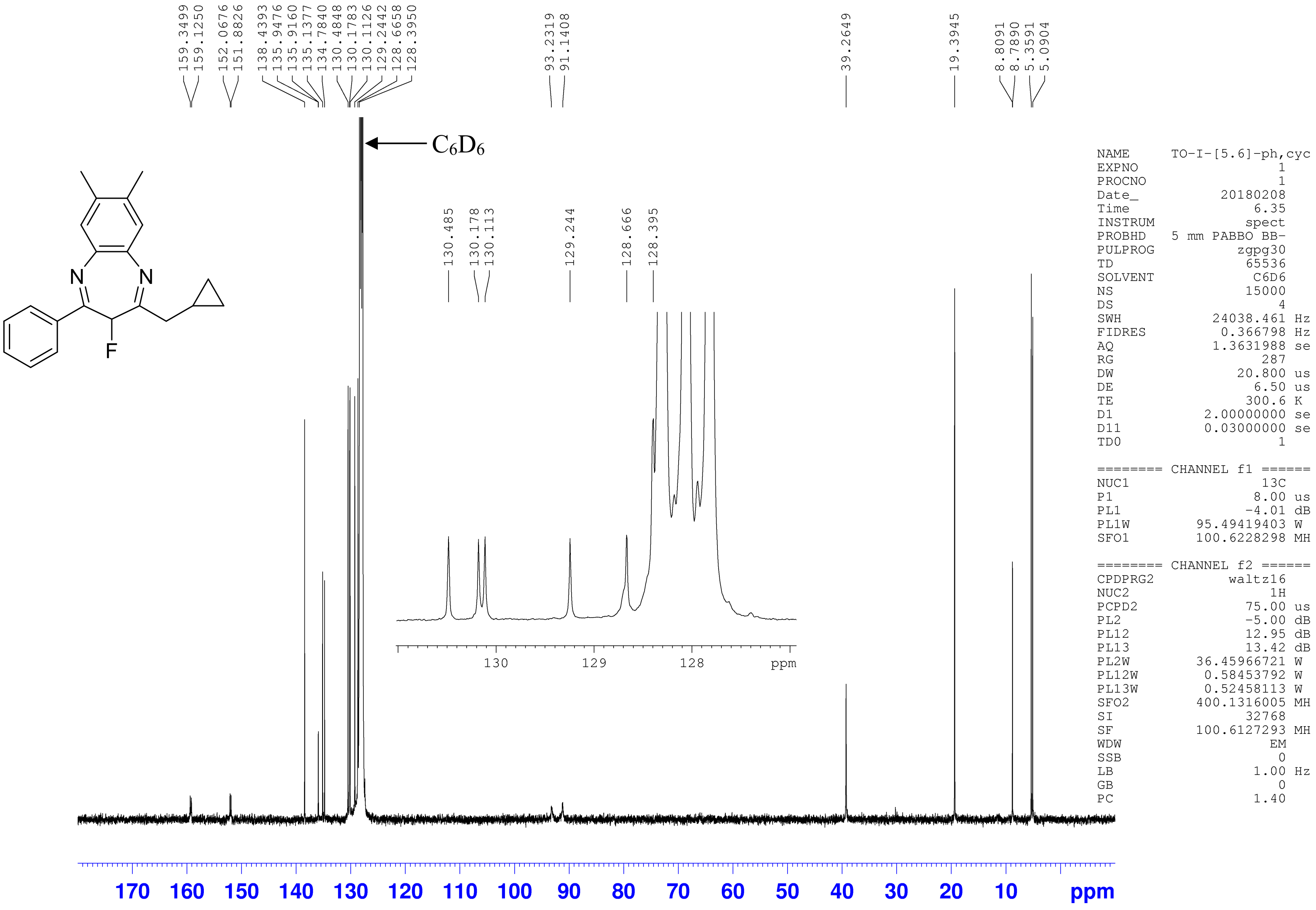

^{1}H NMR spectrum for **5cd** (C_6D_6)

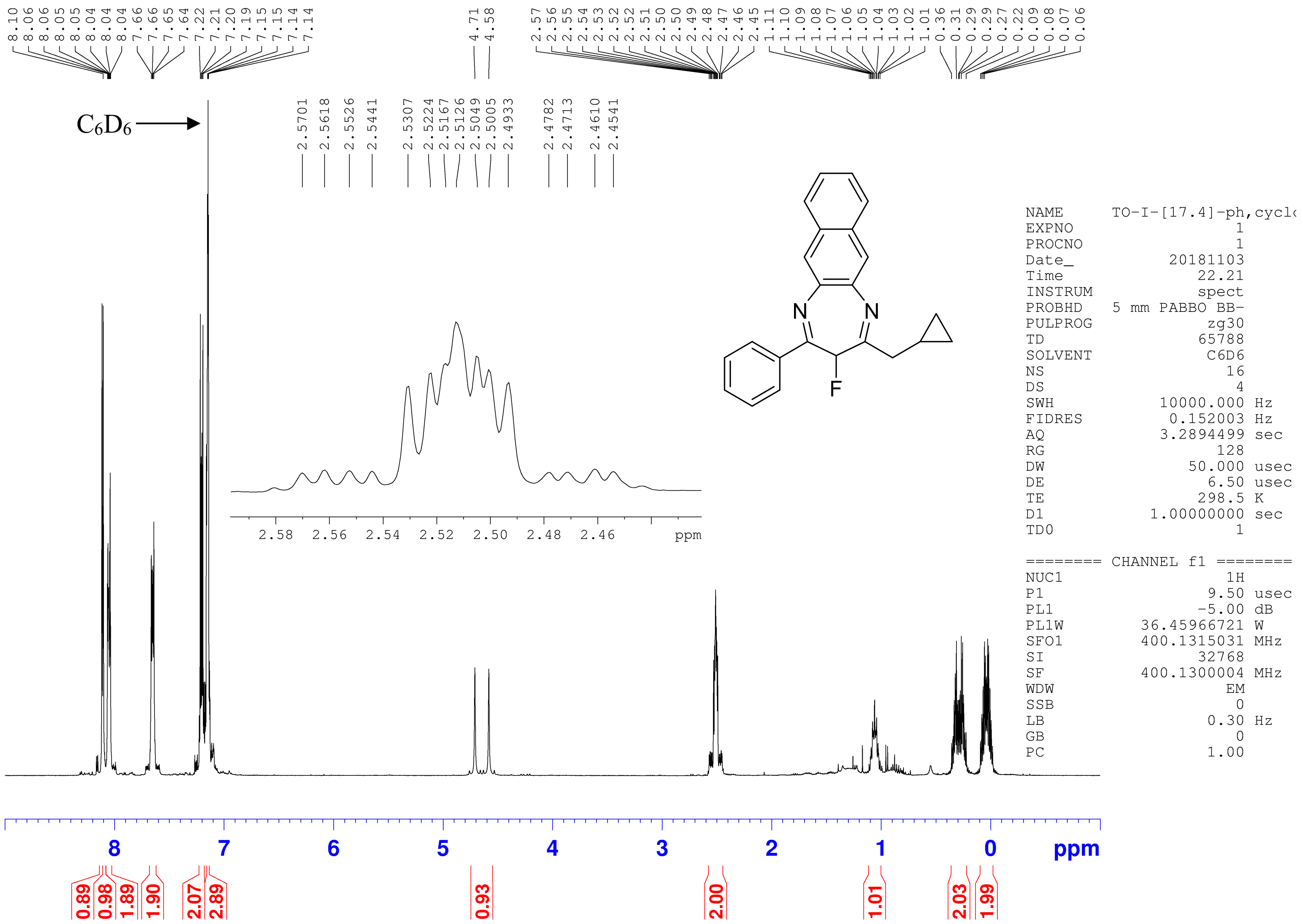

ACS Org. Inorg. Au **2026**, *6*, doi.org/10.1021/acsorginorgau.5c00116

$^{13}C\{^{1}H\}$ NMR spectrum for **5cd** (C_6D_6)

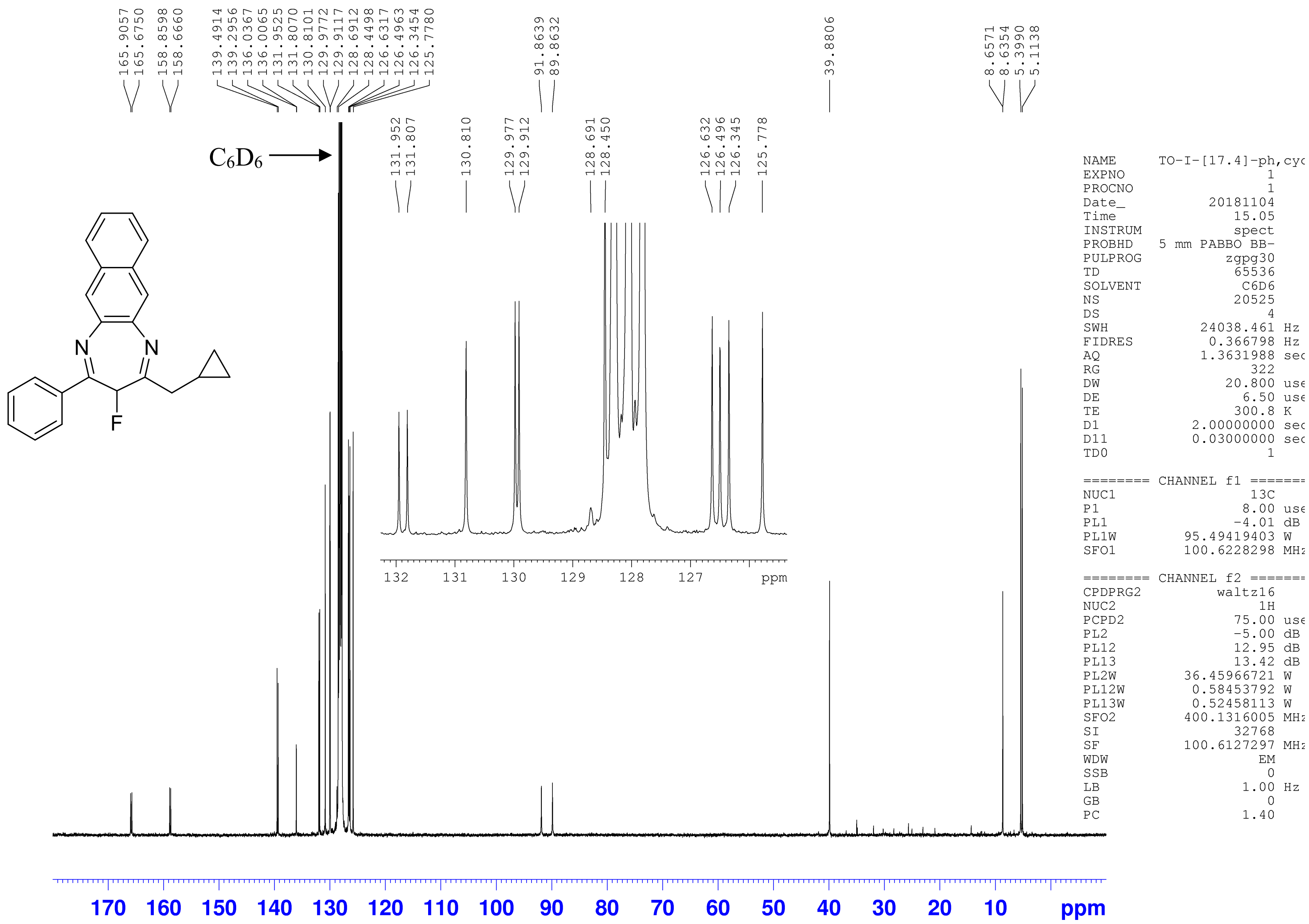

Expansions of ^{1}H NMR cyclopropyl signals of **5ca**, **5cb**, and **5cd**.

5ca

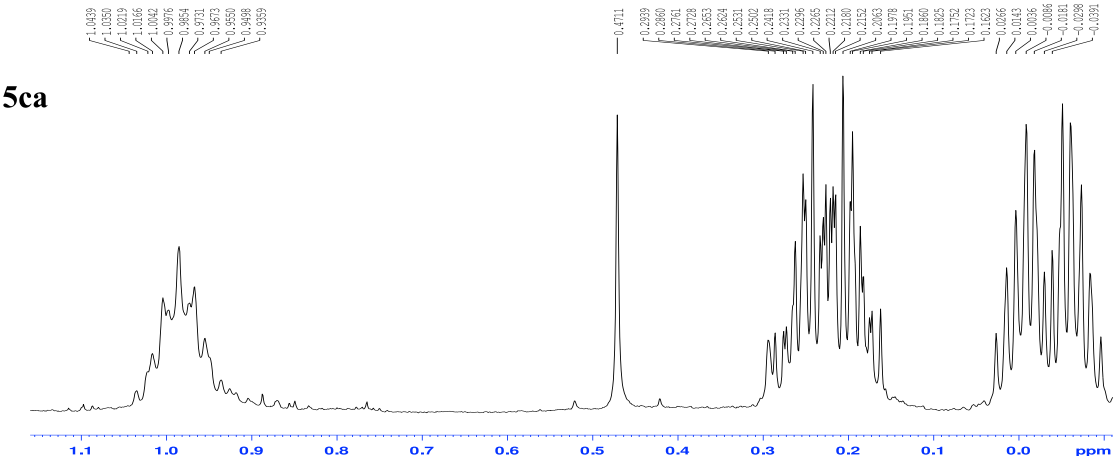

5cb

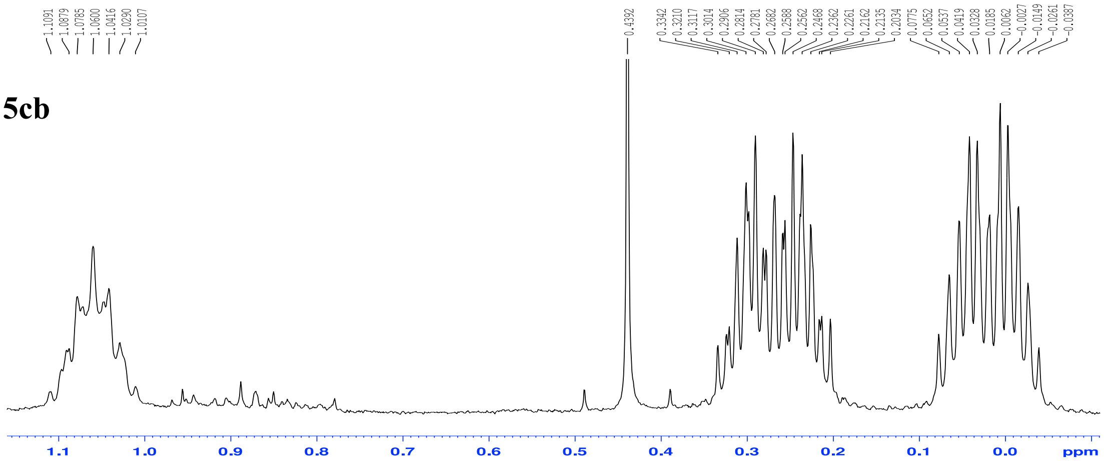

5cd

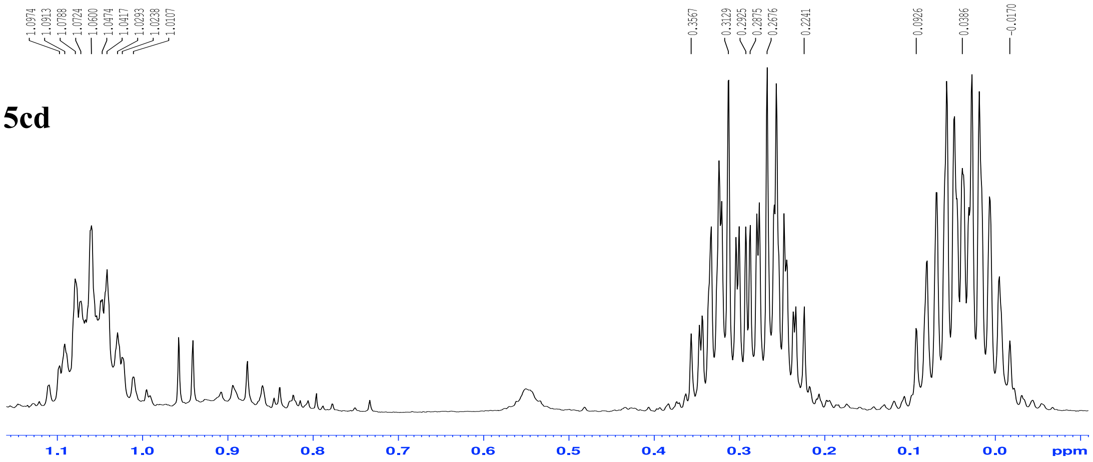

^{1}H NMR spectrum for **5db** (C_6D_6)

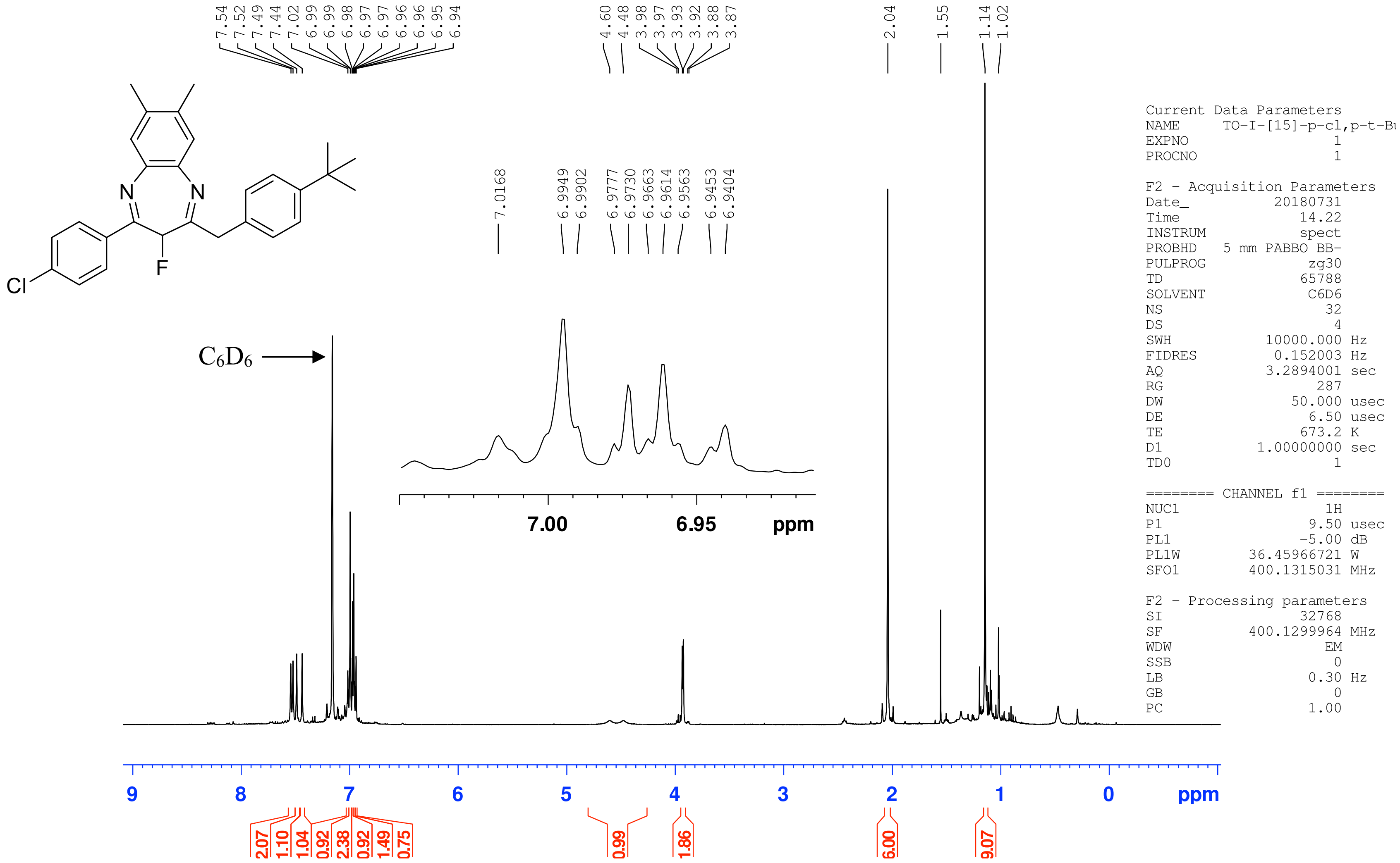

$^{13}C\{^{1}H\}$ NMR spectrum for **5db** (C_6D_6)

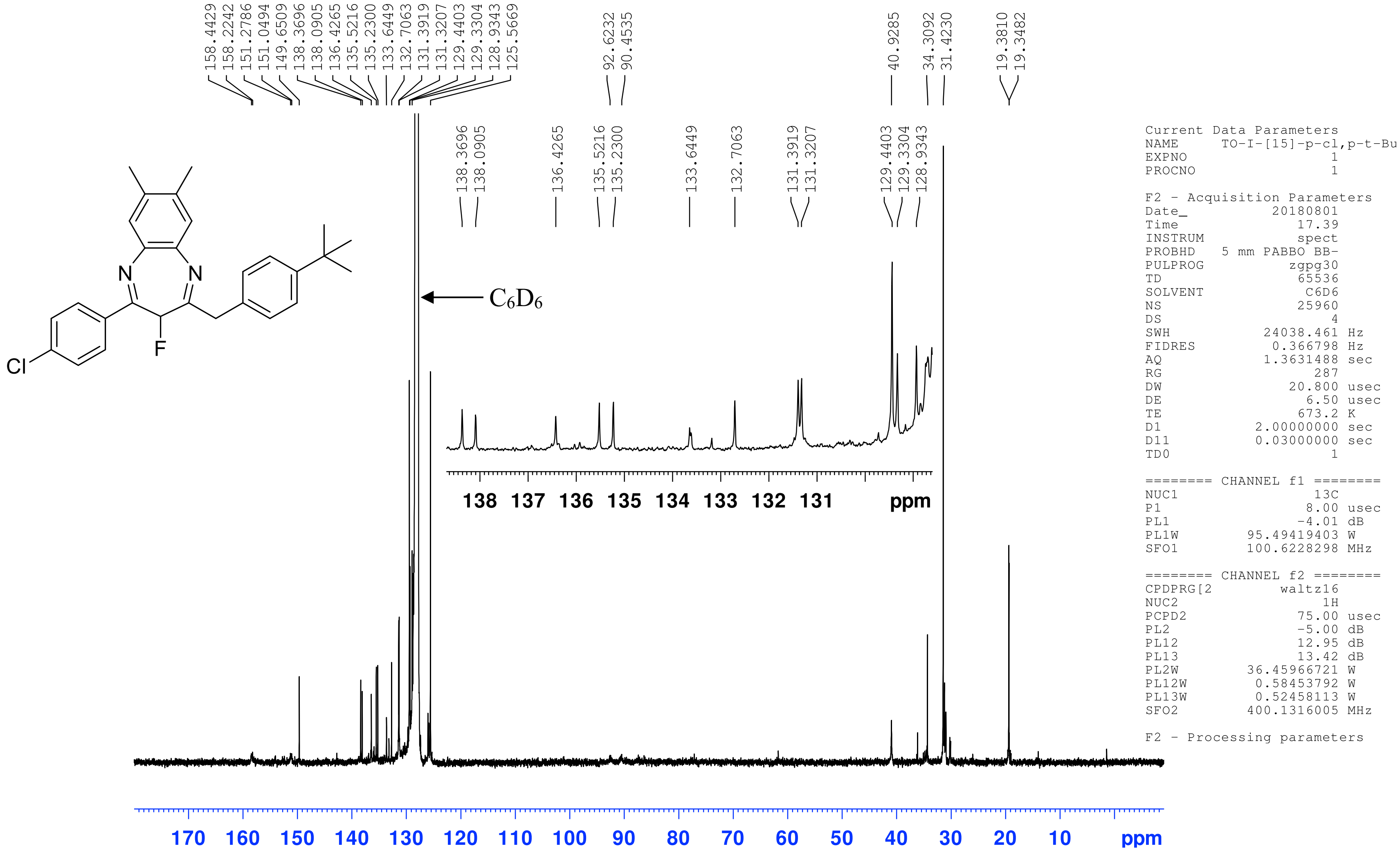

^{1}H NMR spectrum for **8aa** (C_6D_6)

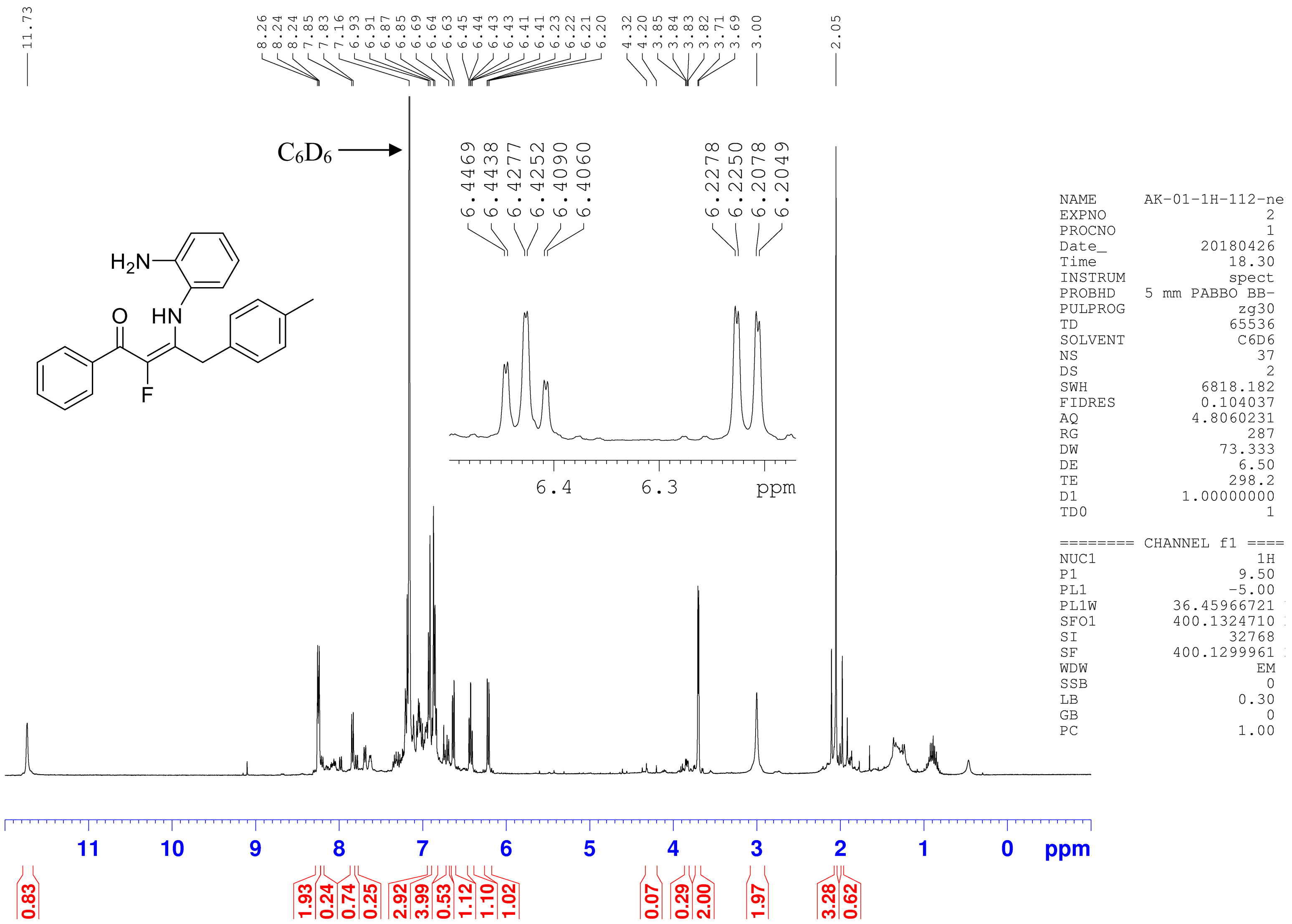

ACS Org. Inorg. Au **2026**, *6*, doi.org/10.1021/acsorginorgau.5c00116

$^{13}C\{^1H\}$ NMR spectrum for **8aa** (C_6D_6)

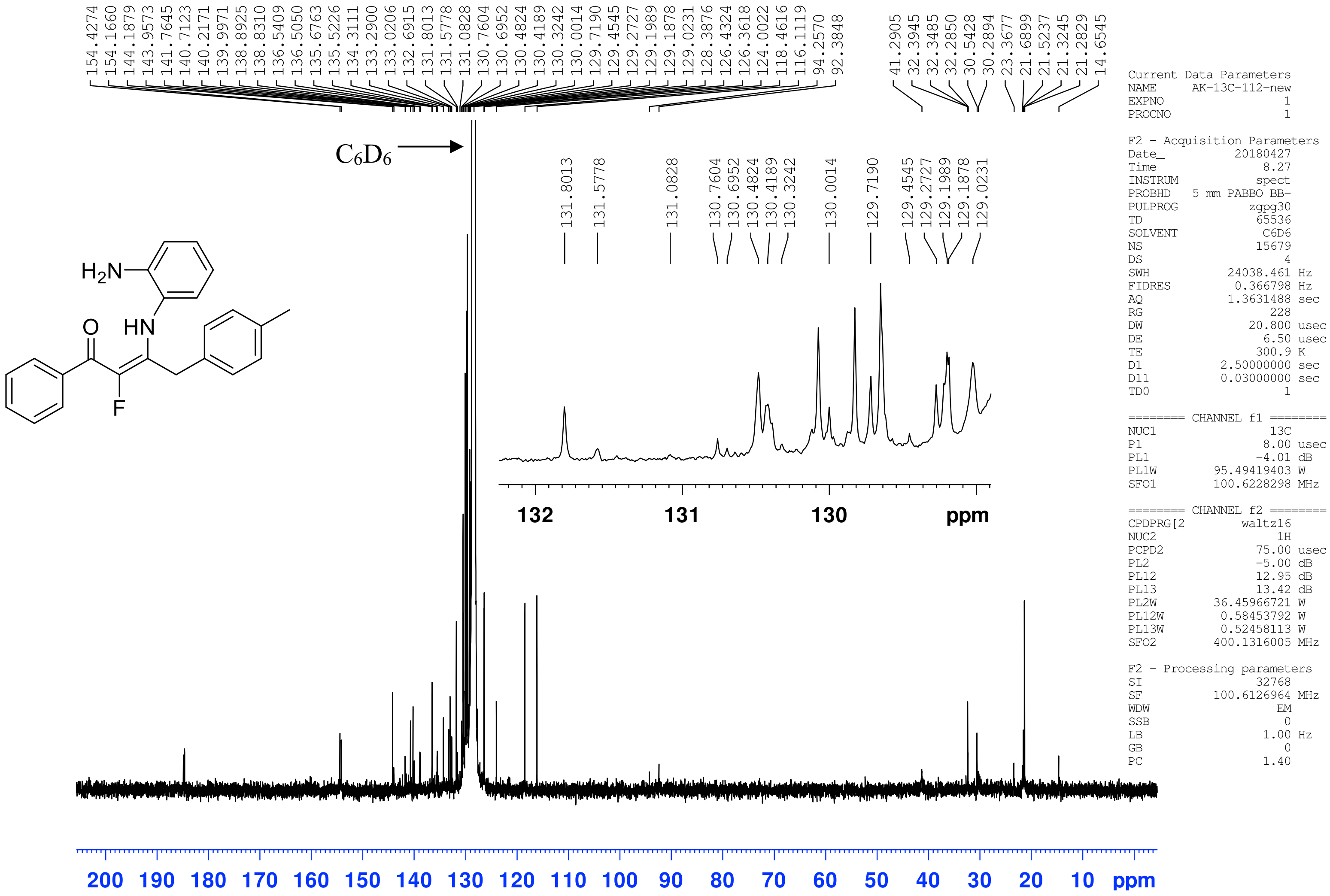


```
Current Data Parameters
NAME     AK-13C-112-new
EXPNO                 1
PROCNO                1

F2 - Acquisition Parameters
Date_          20180427
Time               8.27
INSTRUM           spect
PROBHD   5 mm PABBO BB-
PULPROG         zgpg30
TD                65536
SOLVENT            C6D6
NS                15679
DS                    4
SWH           24038.461 Hz
FIDRES         0.366798 Hz
AQ            1.3631488 sec
RG                  228
DW               20.800 usec
DE                 6.50 usec
TE                300.9 K
D1           2.50000000 sec
D11          0.03000000 sec
TD0                   1

======== CHANNEL f1 ========
NUC1                13C
P1                 8.00 usec
PL1               -4.01 dB
PL1W        95.49419403 W
SFO1       100.6228298 MHz

======== CHANNEL f2 ========
CPDPRG[2        waltz16
NUC2                 1H
PCPD2             75.00 usec
PL2               -5.00 dB
PL12              12.95 dB
PL13              13.42 dB
PL2W        36.45966721 W
PL12W        0.58453792 W
PL13W        0.52458113 W
SFO2       400.1316005 MHz

F2 - Processing parameters
SI                32768
SF         100.6126964 MHz
WDW                  EM
SSB                   0
LB                 1.00 Hz
GB                    0
PC                 1.40
```

Crystallography for 5aa: Details of X-ray Data Collection and Reduction. The crystal with dimension of 0.090×0.127×0.267 mm was affixed to a polyimide loop by Paratone N oil and cooled down in a stream of liquid nitrogen. X-ray data were collected on a Bruker APEX II CCD diffractometer at 100K using phi and omega scans with graphite monochromatic Cu Mo $K\alpha$ (λ = 1.54178 Å) radiation. Data sets were corrected for Lorentz and polarization effects as well as absorption. The criterion for observed reflections is $I > 2\sigma(I)$. Lattice parameters were determined from least-squares analysis and reflection data. Empirical absorption corrections were applied using SADABS.[S1] Structures were solved by direct methods and refined by full-matrix least-squares analysis on F^2 using X-SEED[S2] equipped with SHELXS[S3]. All non-hydrogen atoms were refined anisotropically by full-matrix least-squares on F^2 using the SHELXS program. The data-collection, processing, and refinement statistics are given in Table S1.

Table S1. The data-collection, processing, and refinement statistics for **5aa**.

**Compound**	**5aa**
**Crystal data**	
CCDC	2092208
Chemical formula	$C_{23}H_{19}FN_2$
Formula weight	342.40
Crystal system	monoclinic
Space group	$P2_1/c$
Temperature (K)	100(2)
a [Å]	13.1404(4)
b [Å]	11.0107(3)
c [Å]	12.8880(4)
V [Å^3]	1763.26(9)
Z	4
d_{calc} [g/cm^3]	1.290
Crystal dimensions [mm]	0.090×0.127×0.267
Radiation type	CuKα
μ [mm^{-1}]	0.663
**Data collection**	
Reflections measured	25964
Range/indices (h, k, l)	-15, 15; -12, 13; -15, 15
θ (max, min) [°]	68.22, 3.56
Total no. of unique data	3223
No. of observed data, I > 2σ(I)	2628
No. of variables	235
R_{int}	0.0486
**Refinement**	
R [$F^2 > 2\sigma(F^2)$]	0.0377
$wR(F^2)$	0.0990
S	1.052
No. of reflections	3223
No. of parameters	235
H-atom treatment	H-atom parameters restrained
Δρ (min, max), e/Å^3	-0.250, 0.417

S1) G. M. Sheldrick, SADABS and TWINABS—Program for Area Detector Absorption Corrections, University of Göttingen, Göttingen, Germany, 2010.
S2) L. J. Barbour, *J. Supramol. Chem*., **2001**, *1*, 189.
S3) G. M. Sheldrick, *Acta Crystallogr., Sect. A: Fundam. Crystallogr.*, **2008**, *64*, 112.

Figure S1. Packing pattern viewed along *a*-axis (top) and interactions (bottom) for the **5aa** crystal.

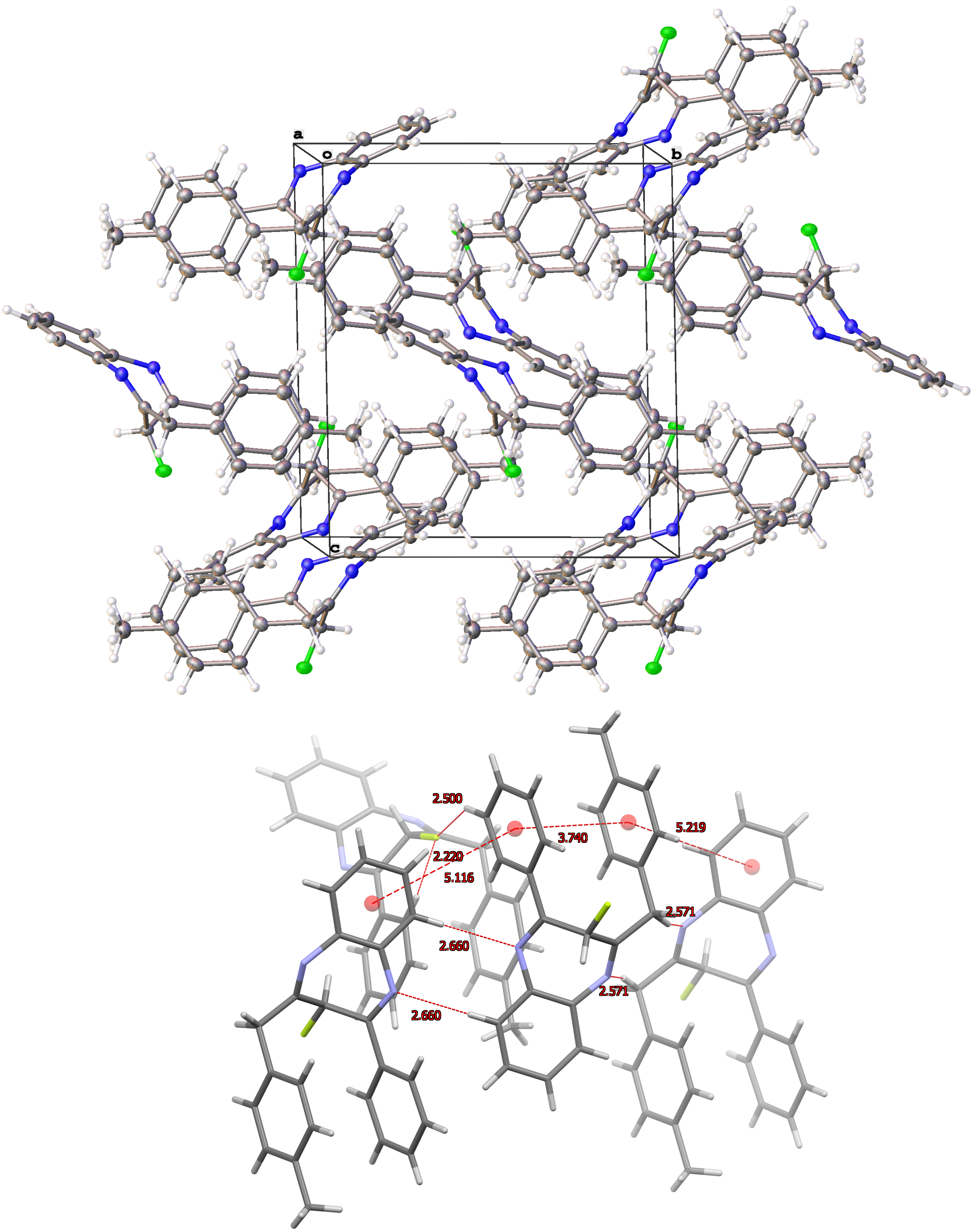

In the crystal packing, adjacent molecules are linked by weak hydrogen bonds N1•••H9–C9 and N5•••H18B–C18, and T-shaped π-stacking (centroid–centroid distances 5.116 Å and 5.219 Å) is also observed between symmetric molecules. Another element connecting these molecules is the F1•••H14–C14 hydrogen bond.
