# Supplementary material for: Non-Catalyzed Cascade Double Imination Reaction of 2‑Fluoro-alk-3-yn-1-ones: Sustainable Synthesis of 3‑Fluoro-1,5-benzodiazepines
Source: ACS Org Inorg Au. 2026 Jan 30;6(2):237–47. doi: 10.1021/acsorginorgau.5c00116 (PMC13047452; doi:10.1021/acsorginorgau.5c00116)
Supplement: Supplementary file 2 [file gg5c00116_si_002.zip › Figure_6/Final_data.pdf]

Species	G, a.u.	Pathway	Species	G-G(7), kcal/mol
5	-1096.413719	A	A-TS1	53.95
7	-1172.798139		8	-6.89
8	-1172.809116		A-TS2	59.61
9	-1096.403074		A-INT	0.36
10	-1096.402845		A-TS3	52.18
11	-1172.805866		9 + H2O	-6.04
H2O	-76.404685		A-TS4 + H2O	61.19
A-INT	-1172.797571			
B-INT	-1172.799260	B	B-TS1	31.51
C-INT	-1172.804791		B-INT	-0.70
A-TS1	-1172.712164		B-TS2	48.39
A-TS2	-1172.703151		10 + H2O	-5.89
A-TS3	-1172.714981		B-TS3 + H2O	48.52
A-TS4	-1096.295941			
B-TS1	-1172.747917	C	C-TS1	57.91
B-TS2	-1172.721032		11	-4.85
B-TS3	-1096.316127		C-TS2	29.03
C-TS1	-1172.705857		C-INT	-4.17
C-TS2	-1172.751870		C-TS3	45.63
C-TS3	-1172.725423			
			5 + H2O	-12.72
